# Autophagic flux blockade under hypocapnia reveals CO_2_-sensitive regulation of autophagy-lysosome homeostasis

**DOI:** 10.1242/bio.062414

**Published:** 2026-06-30

**Authors:** Naghmana Ashraf, Zhen Sun, Jeanine L. Van Nostrand

**Affiliations:** ^1^ Department of Molecular and Cellular Biology, Baylor College of Medicine, Houston, TX 77030, USA; ^2^ Dan L Duncan Comprehensive Cancer Center, Baylor College of Medicine, Houston, TX 77030, USA

**Keywords:** Autophagy, Carbon dioxide, Hypocapnia, Lysosome, mTOR, TFE3

## Abstract

Hypocapnia, a reduction in partial pressure of carbon dioxide (CO_2_), commonly occurs in clinical contexts such as mechanical ventilation, panic disorder, and brain injury, yet its impact on cellular homeostasis remains poorly understood. Given the central role of autophagy in stress adaptation, we investigated how low CO_2_ influences autophagic flux and lysosomal function. We found that hypocapnia induces autophagosome accumulation while impairing cargo degradation, indicating a blockade in autophagic flux. This response was accompanied by increased lysosome biogenesis but, paradoxically, reduced autophagosome-lysosome fusion and lysosomal proteolytic activity. Mechanistically, hypocapnia promoted TFE3 dephosphorylation and nuclear translocation, driving transcriptional activation of lysosomal genes. Concurrently, suppressed AMPK activity and sustained mTOR signaling revealed a unique metabolic state that uncouples energy stress from canonical autophagy control. As such, inhibition of both mTORC1 and mTORC2 was sufficient to restore autophagic flux. Notably, increased pH was not sufficient to drive this program. These findings identify hypocapnia as a previously unrecognized modulator of autophagy that disrupts autophagosome-lysosome fusion and terminal degradation, positioning CO_2_ tension as a critical regulator of cellular stress responses.

## Teaser

**Summary:** Low carbon dioxide impairs autophagic flux by uncoupling lysosome abundance from degradation, revealing CO_2_ tension as a key metabolic signal influencing cellular stress responses and highlighting how experimental conditions can shape biological outcomes.

## INTRODUCTION

Macroautophagy is a fundamental cellular process responsible for the degradation and recycling of damaged organelles, misfolded proteins, and other cytoplasmic material. This catabolic pathway proceeds through the formation of autophagosomes, which fuse with lysosomes to form autolysosomes where degradation occurs. Autophagy plays a key role in stress adaptation, energy homeostasis, immunity, and neuroprotection, and its dysregulation is implicated in a wide range of pathologies including neurodegeneration, cancer, and metabolic diseases (Mizushima et al., 2008; Galluzzi et al., 2017).

Autophagy is not only a stress-response pathway but also a major protein- and organelle-quality control system. By delivering damaged proteins, protein aggregates, and dysfunctional organelles to lysosomes, autophagy maintains proteostasis and prevents accumulation of cytotoxic material (Klionsky et al., 2007; Dikic and Elazar, 2018; Levine and Kroemer, 2019; Mizushima and Komatsu, 2011). This is particularly important in metabolically active or stress-exposed cells, where disruption of autophagic flux can lead to defective protein turnover, impaired mitochondrial quality control, and accumulation of undegraded cargo (Klionsky et al., 2007; Dikic and Elazar, 2018; Pickles et al., 2018).

Lysosomes serve as the terminal degradative compartment in this pathway, and their function depends on coordinated regulation of acidification, hydrolase maturation, vesicle fusion, and nutrient-sensing pathways (Saftig and Klumperman, 2009; Ballabio and Bonifacino, 2020; Perera and Zoncu, 2016). Thus, changes in cellular metabolism or acid–base balance can disrupt not only autophagosome formation but also lysosomal proteolysis and autophagic completion (Ballabio and Bonifacino, 2020; Mindell, 2012). Carbon dioxide (CO_2_) is well positioned to influence these processes because it directly participates in cellular acid–base chemistry through the CO_2_/HCO_3_^−^ buffering system and can thereby alter intracellular and organellar pH (Laffey and Kavanagh, 2002; Roos and Boron, 1981; Casey et al., 2010). Consistent with this, emerging evidence suggests that CO_2_ levels alter intracellular pH, mitochondrial dynamics, and energy metabolism (Casey et al., 2010; Onishi et al., 2012; Vohwinkel et al., 2011; Rivers and Meininger, 2023), all of which are intimately linked to autophagy regulation. Moreover, transcription factors such as TFE3 and TFEB, which orchestrate lysosomal biogenesis and autophagy-related gene expression, are sensitive to metabolic and stress cues that may be influenced by CO_2_ availability (Napolitano and Ballabio, 2016; Settembre et al., 2012). Despite these established links between pH, metabolic signaling, and autophagy, the role of CO_2_ in directly modulating autophagic regulation remains poorly understood.

Hypocapnia, defined as a reduction in arterial CO_2_ partial pressure, commonly arises in clinical settings such as mechanical ventilation, panic disorder, stroke, and traumatic brain injury (Laffey and Kavanagh, 2002; Deng et al., 2020). Despite its prevalence, the cellular consequences of hypocapnia remain poorly understood, particularly with respect to intracellular catabolic and signaling pathways. While elevated CO_2_ levels have been shown to suppress autophagosome formation in macrophages through regulation of Beclin-1 (Casalino-Matsuda et al., 2015), it remains unclear whether reduced CO_2_ elicits reciprocal or distinct effects on autophagy regulation. Notably, studies of hypocapnia have largely focused on vascular or systemic acid–base effects, with comparatively little attention paid to potential cell-intrinsic responses such as protein quality control (Laffey and Kavanagh, 2002; Curley et al., 2016; Nin et al., 2017). Whether hypocapnia perturbs autophagosome turnover, lysosomal function, or autophagic flux has not been systemically examined, representing an important gap in our understanding of how CO_2_ tension influences cellular homeostasis (Casalino-Matsuda et al., 2015; Curley et al., 2016). Defining this relationship is important because impaired autophagic flux can lead to autophagosome accumulation without productive degradation (Klionsky et al., 2007; Mizushima and Levine, 2020), a phenotype likely to be detrimental in tissues vulnerable to hypocapnia-associated stress, including the brain and lung (Laffey and Kavanagh, 2002; Puyal et al., 2013). To address this gap, we investigated the effects of sustained hypocapnia on autophagy and lysosomal dynamics using cellular models exposed to low CO_2_ conditions.

## RESULTS

### Hypocapnia induces autophagy while blocking autophagic flux

**Fig. 1.**
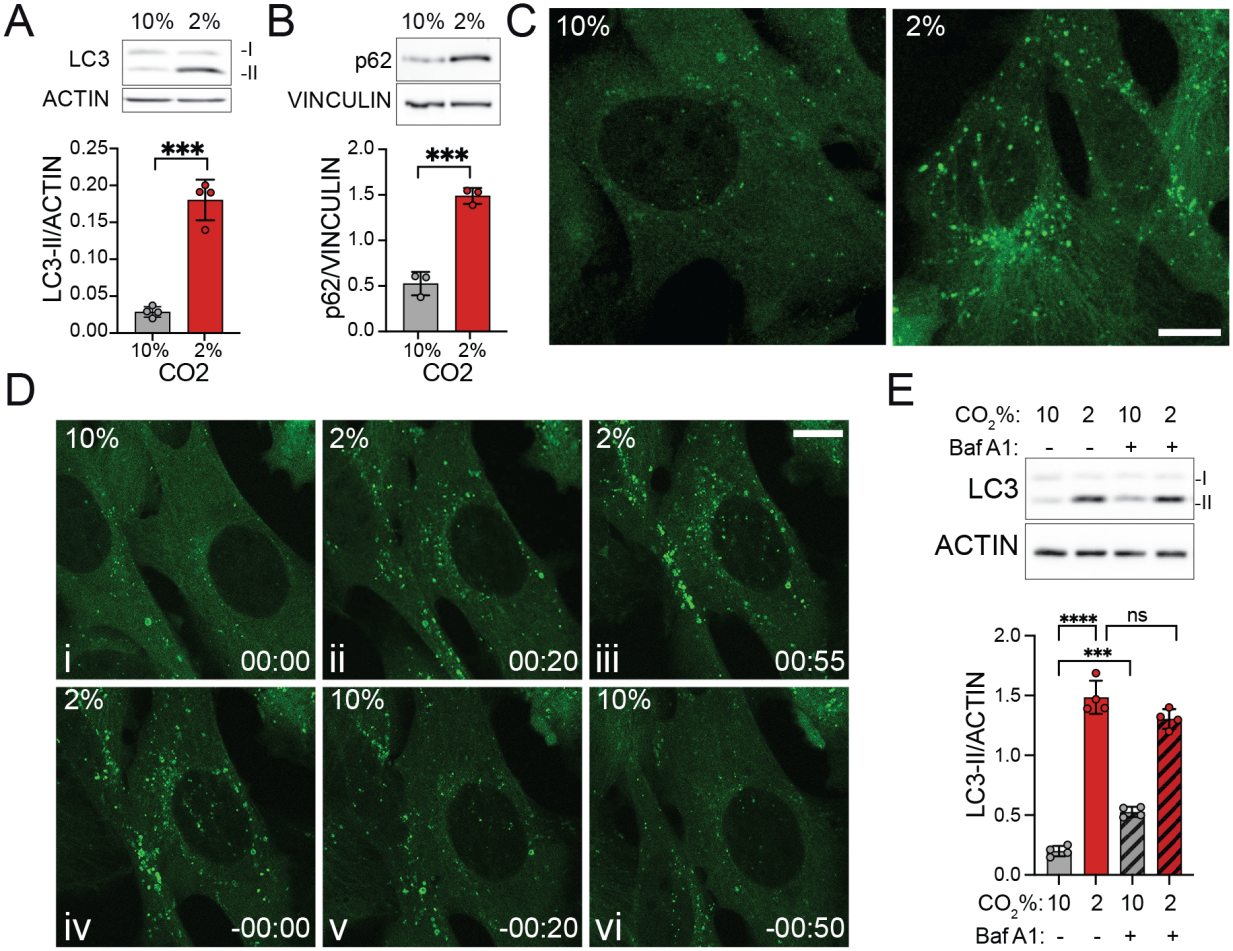
**Assessment of autophagy markers and flux under different CO_2_ conditions.** (A,B) Immunoblot of LC3-II (A) and p62 (B) protein levels in cells cultured under 2% CO_2_ compared to 10% CO_2_. Bottom: quantification relative to ACTIN (*n*=4) or VINCULIN (*n*=3; ***P<0.001, ****P<0.0001; Welch's two-tailed *t*-test). (C) Representative confocal images of cells expressing GFP-LC3 cultured under 10% or 2% CO_2_. Scale bar: 10 μm. (D) Time-lapse imaging of GFP-LC3-expressing cells at 10% CO_2_ (i), switched to 2% CO_2_ (ii-iv) and switched back to 10% (v-vi). Time stamps represent h:min. Scale bar: 10 μm. (E) Immunoblot of LC3-II in cells cultured at 10% or 2% CO_2_ in the presence or absence of 100 nM BafA1 for 2 h. Bottom: Quantification of LC3-II levels relative to ACTIN. (*n*=4; ns, not significant, ***P<0.001, ****P<0.0001; ANOVA with Tukey's multiple comparisons test).

Given the central role of CO_2_ in regulating cellular pH and metabolism, we sought to determine whether reduced CO_2_ levels affect autophagy, a major homeostatic pathway sensitive to cellular stress. T-antigen immortalized mouse embryonic fibroblasts (MEFs) cultured under 2% CO_2_ exhibited a marked increase in autophagosome-associated LC3-II protein levels compared to cells maintained under physiological (10%) CO_2_ (Fig. 1A). This was accompanied by elevated levels of SQSTM1/p62 (Fig. 1B), a cargo receptor degraded via autophagy. Evaluation of a cancer cell line, i.e. human HeLa cells, showed similar increase in LC3-II levels upon culturing at 2% CO_2_, suggesting this phenomenon is a universal response to hypocapnia (Fig. S1A,B). These changes indicate either enhanced autophagosome formation or a block in downstream autophagic turnover.

To further assess autophagosome dynamics, we examined LC3 puncta formation using GFP-LC3-expressing cells. Under 2% CO_2_, cells showed a pronounced accumulation of LC3-positive puncta relative to 10% CO_2_ (Fig. 1C), consistent with increased autophagosome abundance. To directly examine the dynamic response of autophagy to CO_2_ shifts, we performed live-cell time-lapse imaging of GFP-LC3-expressing cells during acute changes in CO_2_ levels (Fig. 1D). Cells were initially imaged under 10% CO_2_ (Fig. 1Di), where few LC3 puncta were observed, consistent with baseline autophagy levels. Upon switching to 2% CO_2_ (Fig. 1Dii), we observed a progressive accumulation of GFP-LC3 puncta, reaching a peak within an hour (Fig. 1Diii-iv), indicating rapid induction of autophagosome formation under hypocapnia conditions. Remarkably, when CO_2_ was restored to 10%, LC3 puncta dramatically cleared (Fig. 1Dv-vi), demonstrating that the autophagy response to hypocapnia is both rapid and reversible. These findings suggest that autophagosome accumulation under low CO_2_ is not due to irreversible autophagy dysfunction but instead reflects an acute, CO_2_-dependent block in autophagic flux that can be relieved upon normalization of CO_2_ levels. This supports a model in which CO_2_ tension acts as a dynamic regulator of autophagy, modulating both autophagosome formation and turnover in response to environmental changes.

To distinguish between increased autophagosome biogenesis and impaired degradation, we treated cells with Bafilomycin A1 (BafA1), a known inhibitor of autophagosome–lysosome fusion that leads to LC3-II accumulation when flux is active. As expected, Bafilomycin A1 significantly increased LC3-II levels in 10% CO_2_ conditions, reflecting normal autophagic flux. However, no further increase was observed in 2% CO_2_ cells treated with Bafilomycin A1 (Fig. 1E), suggesting that LC3-II accumulation under hypocapnia is due to a failure in autophagosome clearance rather than increased autophagic flux.

Together, these data demonstrate that hypocapnia stimulates autophagosome biogenesis – evidenced by increased LC3 puncta and LC3-II accumulation – while simultaneously impairing degradation of autophagic cargo. This suggests a disruption in autophagic flux downstream of autophagosome formation, potentially at the level of autophagosome–lysosome fusion or lysosomal function.

### pH is not sufficient to drive blockage of autophagy

**Fig. 2.**
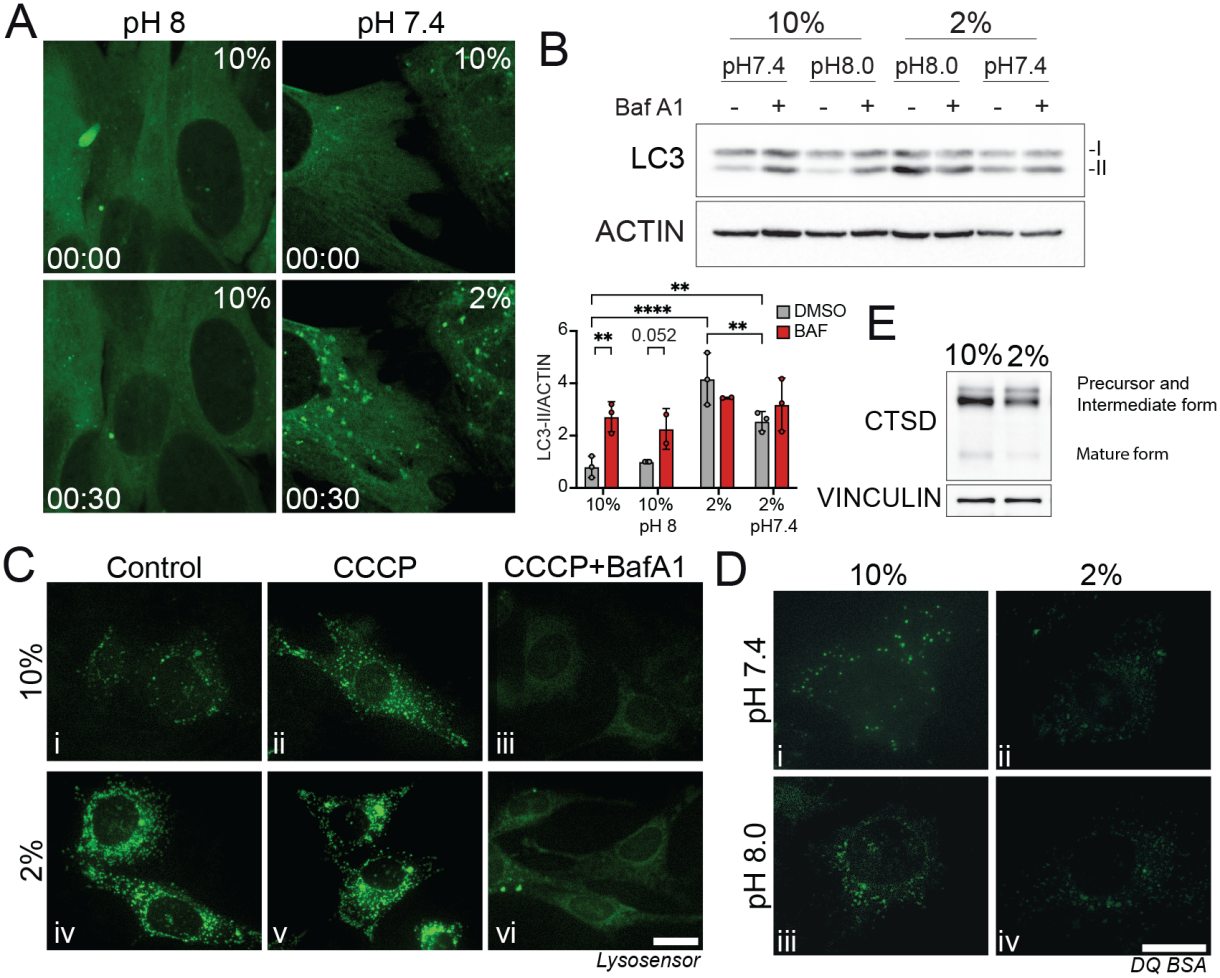
**Analysis of lysosomal function under different pH conditions.** (A) Representative confocal images of GFP–LC3-expressing cells cultured under 10% CO_2_ at pH 8 or 2% at pH 7.4. Time stamps represent h:min. Scale bar: 10 μm. (B) Western blot analysis of LC3 in cells cultured under 2% or 10% CO_2_ at pH 8 or pH7.4 for 4 h. Bottom: Quantification of LC3-II expression relative to ACTIN (Nin et al., 2017; *n*=3; **P<0.01, ****P<0.0001; two-way ANOVA with Fisher LSD test). (C) Lysosensor staining in cells maintained at 2% CO_2_ compared to 10% CO_2_. 20 μM CCCP treatment for 2 h (positive control) and 100 nM Bafilomycin A1 (BafA1) for 2 h (negative control). Scale bar: 20 μm. (D) DQ-BSA (50 μg/ml) in cells cultured under 10% CO_2_ at pH 8 or 2% at pH 7.4. Scale bar: 20 μm. (E) Immunoblot of Cathepsin D (CTSD) showing precursor, intermediate and mature forms under 10% and 2% CO_2_. VINCULIN used as loading control.

A major consequence of reduced CO_2_ levels is a concomitant increase in media pH levels. Previous studies have shown that media alkalinization can influence mTOR signaling and cell survival (Kazyken et al., 2023, 2021). To determine whether increased pH contributes autophagosome accumulation under low CO_2_ conditions, we cultured cells at 10% CO_2_ while adjusting the media pH to 8.0, matching the pH typically observed at 2% CO_2_. Strikingly, cells exposed to pH 8.0 under normocapnic conditions for 30-50 min failed to form LC3 puncta (Fig. 2A), indicating that pH elevation alone is insufficient to drive autophagosome accumulation.

To assess whether pH changes are required for LC3 puncta formation under hypocapnic conditions, we maintained the media at pH 7.4 while switching the cells to 2% CO_2_. Despite the media pH remaining neutral, robust LC3 puncta formation was observed within 30 min at 2% CO_2_. These findings were further supported by western blot analysis of cells maintained at pH 7.4 or pH8.0 under either 10% or 2% CO_2_ levels (Fig. 2B). Consistent with imaging data, increasing the pH at 10% CO_2_ did not alter LC3 levels or affect the response to Bafilomycin A1. In contrast, exposure to 2% CO_2_ resulted in increased LC3 levels and resistance to Bafilomycin regardless of extracellular pH. Notably, LC3 accumulation at lower pH was reduced compared with higher pH, suggesting that pH may modulate autophagy initiation but does not account for the blockade in autophagic flux.

Because changes in CO_2_ levels may influence intracellular acidification independently of extracellular pH, we next assessed lysosomal acidification using LysoSensor Green, a pH-sensitive dye that accumulates in acidic compartments. Cells cultured under 2% CO_2_ exhibited an increased overall LysoSensor signal relative to cells maintained at 10% CO_2_ (Fig. 2C). While the number of LysoSensor-positive puncta and total signal were increased, fluorescence intensity per lysosome was comparable between conditions. This signal was comparable to that induced by CCCP treatment and was abolished by Bafilomycin A1, confirming that the observed fluorescence reflected lysosome-specific acidification. These data indicate that lysosomes remain acidified under hypocapnic conditions.

To directly evaluate lysosomal proteolytic capacity, we used the DQ-BSA assay, which fluoresces upon degradation by active lysosomal enzymes (Frost et al., 2017). Under basal conditions at 10% CO_2_ and normal pH, cells displayed modest DQ-BSA processing (Fig. 2Di). In contrast, cells cultured at 2% CO_2_ in complete media exhibited reduced DQ-BSA fluorescence despite an increased number of acidified lysosomes (Fig. 2Div). Importantly, altering pH at either 10% or 2% did not change the CO_2_-dependent effect on DQ-BSA processing (Fig. 2Dii, iii). As a positive control, glucose- and serum-starved cells at 10% CO_2_ exhibited robust DQ-BSA fluorescence (Fig. S1Ci), which was reduced upon Bafilomycin A1 treatment (Fig. S1Cii). The absence of DQ-BSA processing under 2% CO_2_ indicates that hypocapnia suppresses lysosomal proteolytic activity despite high autophagy induction.

We further examined lysosomal function by assessing maturation of Cathepsin D (CTSD), a lysosomal hydrolase whose processing reflects proper lysosomal acidification and enzyme activation. Conversion of CTSD from its precursor to intermediate and mature forms was reduced under 2% CO_2_ (Fig. 2E), reinforcing the conclusion that lysosomal degradative function is disrupted.

Collectively, these results demonstrate that CO_2_ regulates autophagy through mechanisms that are not solely dependent on extracellular or cytoplasmic pH. The inability of pH elevation alone to recapitulate LC3 puncta formation, together with preserved lysosomal acidification but impaired proteolytic activity under low CO_2_, suggests involvement of additional CO_2_-sensitive metabolic or signaling pathways in the regulation of autophagic flux.

### Hypocapnia induces lysosomal biogenesis via TFE3 activation

**Fig. 3.**
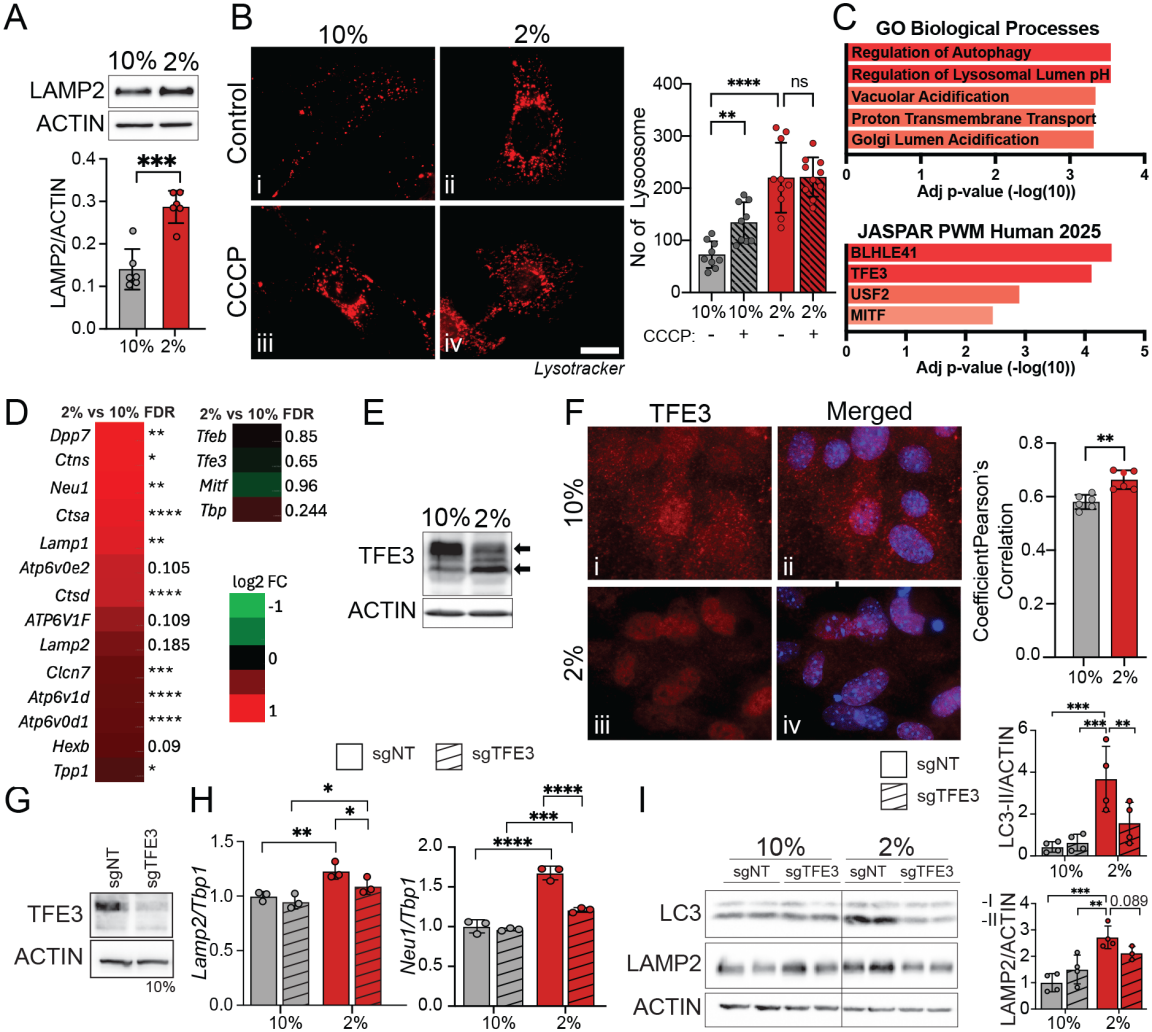
**Lysosomal biogenesis driven by TFE3 under hypocapnic conditions.** (A) Immunoblot of LAMP2 in cells maintained at 2% CO_2_ relative to 10% CO_2_. Quantification of LAMP2 expression relative to ACTIN (*n*=6; ***P<0.001; Welch's two-tailed *t*-test). (B) Lysotracker staining in cells maintained at 2% CO_2_ (ii) compared to 10% CO_2_ (i). 2 h of 20 μM CCCP treatment used as a positive control for increased lysosomes (iii, iv). Scale bar: 20 μm. Right: quantification of lysosome number from lysotracker staining in cell maintained at 2% CO_2_ compared to 10% CO_2_, with or without 2 h of 20 μM CCCP treatment (ns, not significant, **P<0.01, ****P<0.0001; two-way ANOVA with Fisher LSD test). (C) GO term enrichment of (top) biological processes and (bottom) transcription factors upregulated upon hypocapnic condition. (D) Heatmap of TFE3 target genes upregulated by hypocapnic conditions. (E) Immunoblot of TFE3 band shifting in cell maintained at 2% CO_2_ relative to 10% CO_2_. (F) Cell immunofluorescence of TFE3 (red) and nuclei (blue) in cells maintained at 2% CO_2_ (iii,iv) compared to 10% CO_2_ (i,ii). Scale bar: 20 μm. Right: quantification of Coefficient Pearson's Correlation between TFE3 immunofluorescence and DAPI staining. (**P<0.01; Welch's two-tailed *t*-test). (G) Immunoblot of TFE3 knockdown in cells cultured at 10% with gRNA targeting non-targeting (sgNT) or TFE3 loci (sgTFE3). (H) qRT-PCR analysis of lysosomal genes (*Lamp2*, *Neu1*) in cells maintained at 2% CO_2_ upon control or TFE3 knockdown. Data normalized to *Tbp1*. (*n*=3; *P<0.05, **P<0.01, ***P<0.001, ****P<0.0001; two-way ANOVA with Fisher LSD test). (I) Immunoblot of LC3 and LAMP2 in cells cultured at 2% or 10% with control (sgNT) or TFE3 (sgTFE3) knockdown. Consolidated lanes denoted by solid line. Right: quantification of LC3-II or LAMP2 levels relative to ACTIN. (*n*=3; **P<0.01, ***P<0.001; two-way ANOVA with Fisher LSD test).

Given that hypocapnia led to autophagosome accumulation but impaired autophagic flux, we next investigated whether changes in lysosomes underlie this disruption. Because lysosomes are essential for the final degradation step of autophagy (Galluzzi et al., 2017) and we observed an overall increase in lysosomal acidification, we examined whether low CO_2_ conditions affect lysosomal abundance. Western blot analysis of MEFs maintained at 2% or 10% CO_2_ revealed significantly higher levels of the lysosomal marker LAMP2 under hypocapnic conditions, indicating increased lysosome abundance (Fig. 3A). The increase was independently confirmed by LysoTracker staining, which demonstrated a higher number of lysosomes in cells maintained at 2% CO_2_ (Fig. 3B). To further validate this effect, we treated cells with the mitochondrial uncoupler and autophagy inducer carbonyl cyanide m-chlorophenylhydrazone (CCCP). In cells cultured at 10% CO_2_, CCCP treatment resulted in a marked increase in lysosome number (Fig. 3B). In contrast, CCCP did not further increase lysosomal abundance in cells maintained at 2% CO_2_ (Fig. 3B). These observations suggest that low CO_2_ is sufficient to induce lysosomal abundance to levels comparable to those achieved by CCCP treatment under normocapnic conditions.

To explore the mechanism driving this increase in lysosomes, we performed RNA sequencing of cells cultured at 10% or 2% CO_2_. We identified 221 genes that were significantly upregulated and 368 genes that were significantly downregulated (fold change>twofold, adjusted *P*-value <0.05) under hypocapnic conditions. Gene Ontology analysis of upregulated genes revealed significant enrichment for pathways involved in autophagy and lysosomal acidification (Fig. 3C). Analysis of enriched transcriptional regulators identified the lysosomal biogenesis transcription factor TFE3 as a key candidate (Fig. 3C). Consistent with this, multiple lysosomal genes known to be TFE3 targets were upregulated in cells cultured at 2% CO_2_ (Fig. 3D)*.* Notably, mRNA expression levels of TFE3 and its closely related family members TFEB and MITF were not altered, suggesting a post-transcriptional regulation. Importantly, although TFEB and MiTF have overlapping transcriptional targets with TFE3, their markedly lower mRNA expression levels (9.3-fold and 17-fold, respectively) suggest that TFE3 is the primary driver of lysosomal biogenesis in this context.

Accordingly, western blot analysis of TFE3 revealed aa increase in dephosphorylated form of TFE3 under hypocapnic conditions (Fig. 3E). Dephosphorylated TFE3 is known to be able to translocate into the nucleus, where it drives transcription of lysosomal biogenesis genes. We therefore assessed TFE3 localization by immunofluorescence. Under normocapnic conditions, TFE3 was predominantly cytoplasmic, whereas cells maintained at 2% CO_2_ displayed near-exclusive nuclear localization of TFE3 (Fig. 3F), consistent with its activation. Together, these data indicated that low CO_2_ promotes lysosomal biogenesis through TFE3-dependent transcriptional programs.

To directly assess the role of TFE3 in mediating these transcriptional changes, we generated TFE3 knockdown MEFs using CRISPR and confirmed efficient depletion by western blot under standard 10% CO_2_ culture conditions (Fig. 3G). We then examined the mRNA expression of two well-established TFE3 targets, *Neu1* and *Lamp2*. Under normocapnic conditions, TFE3 knockdown had no effect on the basal expression of either gene. However, whereas control cells culture at 2% CO_2_ showed a significant induction of *Neu1* and *Lamp2* expression, this response was significantly attenuated upon TFE3 knockdown (Fig. 3H).

We next analyzed LC3 and LAMP2 protein levels by western blot following culture at 2% CO_2_. Consistent with the transcriptional data, no difference in LC3-II or LAMP2 levels were observed between control and TFE3 knockdown cells at 10% CO_2_. In contrast, exposure to 2% CO_2_ led to a robust increase in LC3-II and LAMP2 levels in control cells, whereas this increase was largely suppressed in TFE3-depleted cells, yielding protein levels comparable to those observed under normocapnic conditions (Fig. 3I). Together, these data demonstrate that TFE3 activation is a key driver of hypocapnic-induced lysosomal biogenesis and contributes significantly to the associated autophagic response.

### Hypocapnia-induced autophagic flux blockade stems from defective vesicle fusion, which is rescued by nutrient stress

**Fig. 4.**
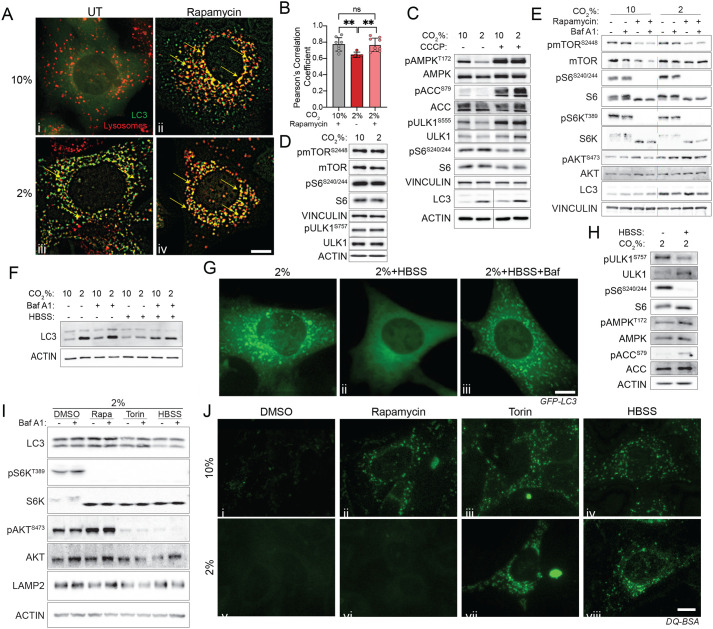
**Assessment of autophagosome–lysosome fusion, metabolic status, and nutrient stress response under different CO_2_ conditions.** (A) Representative images showing colocalization of LC3 (green) and lysosomes (red) in cells cultured under (i) 10% CO_2_, (ii) 10% CO_2_+Rapamycin, (iii) 2% CO_2_ or (iv) 2% CO_2_+Rapamycin. Yellow indicates merged signal (colocalized vesicles); arrows highlight LC3–lysosome overlap. Scale bar: 10 μm. (B) Quantification of Pearson's correlation coefficient for LC3–lysosome colocalization across indicated conditions (ns, not significant, **P<0.01; two-way ANOVA with Fisher LSD test). (C) Immunoblot of AMPK activity under 10% and 2% CO_2_ in presence or absence of 20 μM CCCP for 2 h. ACTIN serves as loading control. Consolidated lanes denoted by solid line. (D) Immunoblot of mTORC1 activity under 10% and 2% CO_2_. VINCULIN serves as loading control. (E) Immunoblot analysis of mTORC1 signaling under 2% or 10% CO_2_ upon treatment with Rapamycin for 6 h with or without Bafilomycin A for 2 h. VINCULIN serves as loading control. (F) Immunoblot of LC3-II under different CO_2_ conditions with or without Bafilomycin A1 and/or HBSS treatment. ACTIN serves as loading control. (G) Representative GFP-LC3 images of cells under (i) 2% CO_2_, (ii) 2% CO_2_+HBSS, and (iii) 2% CO_2_+HBSS+Bafilomycin A1. Scale bar: 10 μm. (H) Immunoblots showing mTORC1 and AMPK activity in cells cultured under 2% CO_2_ with or without HBSS treatment. ACTIN serves as loading control. (I) Immunoblots showing mTORC1 activity under 2% CO_2_ with Rapamycin (16 h), Torin (16 h), or HBSS (4 h) with or without Bafilomycin A (2 h). ACTIN as loading control. (J) DQ-BSA (50 μg/ml) in cells cultured under 2% or 10% CO_2_ treated with DMSO, Rapamycin (16 h), Torin1 (16 h), or HBSS (4 h). Scale bar: 20 μm.

We next investigated whether the blockade in autophagy flux observed at 2% CO_2_, despite induction of lysosomal biogenesis, reflects defective autophagosome–lysosome fusion. Because basal autophagosome levels are low under normocapnic conditions, we treated cells with the mechanistic target of rapamycin complex I (mTORC1) inhibitor rapamycin to induce autophagy. As expected, rapamycin treatment at 10% CO_2_ promoted robust colocalization of GFP-LC3 puncta with lysosomes (Fig. 4Ai-ii,B) In contrast, despite elevated basal autophagosome levels in cells cultured at 2% CO_2_, we observed significantly reduced spatial overlap between GFP-LC3 and lysosomes under basal conditions (Fig. 4Aiii,B), indicating that while autophagosomes form under hypocapnia, their interaction with lysosomes is impaired.

Because autophagosome–lysosome fusion and lysosomal degradation are energy-dependent processes regulated by the AMPK and mTOR pathways (Mindell, 2012), we next assessed cellular energy status under hypocapnic conditions. Surprisingly, intracellular adenosine triphosphate (ATP) levels were significantly elevated in cells cultured at 2% CO_2_, suggesting that defective autophagic flux occurs despite an apparent energy-replete state (Fig. S1D). Consistent with this, AMPK^T172^ phosphorylation was markedly reduced under hypocapnia, along with decreased phosphorylation of its downstream targets ACC^S79^ and ULK1^S555^ (Fig. 4B), indicating suppression of energy stress signaling. In contrast, mTORC1 activity remained largely unchanged under hypocapnic conditions, as evidenced by persistent phosphorylation of canonical mTORC1 substrates, including S6^S240/244^ and ULK1^S757^ (Fig. 4C).

To investigate whether mTORC1 activity directly contributes to impaired autophagosome-lysosome fusion under hypocapnic conditions, we treated cells with the mTORC1 inhibitor rapamycin. Surprisingly, treatment with rapamycin induced robust colocalization of LC3 and lysosomes at 2% CO_2_ to levels comparable to cells at 10% treated with rapamycin (Fig. 4Aiv). This suggests that mTORC1 activity, although not elevated by hypocapnia, nonetheless prevents efficient fusion of autophagosomes and lysosomes under hypocapnic conditions.

Despite restored autophagosome-lysosome colocalization, rapamycin failed to rescue the autophagic flux observed under hypocapnic conditions, indicating that recovery of fusion alone is insufficient to overcome the autophagic blockade induced by low CO_2_ (Fig. 4E). As expected, rapamycin effectively inhibited mTORC1 signaling, as indicated by reduced P-S6^S240/244^ and P-S6K^T389^ levels. However, rapamycin treatment concomitantly increased mTORC2 signaling, as determined by P-AKT^S473^ (Fig. 4E). Notably, basal P-AKT levels were also higher in untreated cells cultured at 2% CO_2_, suggesting that enhanced mTORC2-AKT signaling may contribute to the persistent impairment of autophagic flux under hypocapnic conditions.

To determine whether nutrient stress and combined mTORC1/mTORC2 inhibition could overcome this block, we treated cells cultured at 2% CO_2_ with Hank's Balanced Salt Solution (HBSS), a nutrient-deprivation medium that robustly activates autophagy. Strikingly, HBSS treatment reduced LC3-II levels and restored responsiveness to Bafilomycin A1 at 2% CO_2_ (Fig. 4F), indicating reactivation of autophagic flux. GFP-LC3 imaging confirmed clearance of LC3 puncta with HBSS, which reaccumulated upon Bafilomycin A1 co-treatment (Fig. 4G), consistent with restored autophagosome–lysosome fusion and cargo turnover. Notably, HBSS reactivated AMPK activity and suppressed mTOR activity (Fig. 3H), supporting a causal role for metabolic signaling and cellular energy status in the hypocapnia-induced autophagy blockade.

Because HBSS inhibits both mTORC1 and mTORC2 while also creating an acidic context, we sought to isolate the role of mTOR signaling independent of pH changes. We therefore evaluated the effects of the pan-mTOR inhibitor Torin1 on autophagic flux and lysosomal function. Torin1 treatment reduced LC3-II levels and restored Bafilomycin A sensitivity under 2% CO_2_, indicating recovery of autophagic flux (Fig. 4I). mTORC1 and mTORC2 inhibition were verified by loss of phosphorylation of S6K^T389^ and AKT^S473^, respectively. Of note, we observed that treatment with rapamycin induced higher P-AKT levels suggesting that mTORC1 partially inhibiting mTORC2 activity, which is relieved with rapamycin treatment. We next evaluated lysosomal proteolytic activity using the DQ-BSA assay. As observed previously, baseline DQ-BSA processing was low at 10% CO_2_ and further suppressed at 2% CO_2_ (Fig. 4J). Rapamycin induced DQ-BSA processing at 10% CO_2_, but failed to do so under hypocapnia, reinforcing that improved fusion alone is insufficient to store lysosomal function. In contrast, both Torin1 and HBSS robustly induced DQ-BSA processing under both normocapnic and hypocapnic conditions, indicating restoration of lysosomal degradative capacity.

**Fig. 5.**
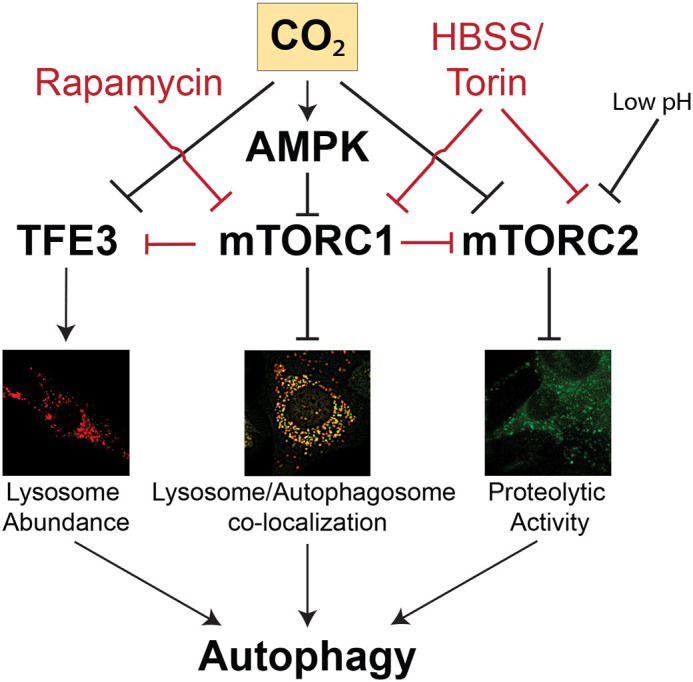
**CO_2_ modulates autophagy.** CO_2_ levels modulates TFE3, AMPK and mTORC1/2 signaling to regulate lysosome abundance, autophagosome-lysosome fusion, and proteolytic function, which collectively impact the rate of autophagy.

Together, these data identify hypocapnia as a regulator of autophagic flux that uncouples autophagosome accumulation from lysosomal degradation (Fig. 5). Low CO_2_ promotes TFE3-driven lysosomal biogenesis while creating a metabolically replete state, marked by elevated ATP levels and suppression of AMPK–ULK1 signaling. Although autophagosome–lysosome fusion is impaired under hypocapnic conditions, restoring fusion alone is insufficient to reestablish flux, as lysosomal proteolytic function remains compromised. Instead, autophagic flux is recovered only upon nutrient stress or pan-mTOR inhibition, demonstrating that CO_2_ tension controls autophagy through coordinated regulation of cellular metabolism, vesicle fusion, and lysosomal function.

## DISCUSSION

Our findings identify hypocapnia as a novel and previously unrecognized disruptor of autophagic flux. Using multiple complementary approaches, we demonstrate that low CO_2_ promotes rapid and reversible autophagosome accumulation while impairing terminal degradation, leading to a dissociation between autophagy initiation and completion. Mechanistically, this uncoupling is associated with a high-ATP, low-AMPK signaling state, sustained mTORC1 activity with enhanced mTORC2 signaling, increased lysosome biogenesis via TFE3 activation, and specific defects in autophagosome–lysosome fusion and proteolysis. Importantly, this block is reversible with nutrient stress, specifically through dual inhibition of mTORC1/2, indicating that CO_2_ tension dynamically regulates autophagic resolution. These findings reveal previously unappreciated links between gas signaling, energy metabolism, and vesicle trafficking.

Autophagy is traditionally regulated by metabolic cues, including glucose deprivation, mTORC1 inhibition, and AMPK activation (Hardie et al., 2012). However, CO_2_ tension has not been widely recognized as an input into autophagic control. Our study challenges this view by showing that hypocapnia induces a pseudo-stressed state in which autophagosome formation is enhanced, yet degradative capacity is suppressed. This paradoxical state is marked by accumulation of LC3-II and p62 and resistance to further LC3-II increase upon Bafilomycin A1 treatment, hallmarks of autophagic flux blockade. Time-lapse imaging confirmed this phenotype is rapid and reversible, indicating a dynamic CO_2_-responsive regulatory mechanism. Importantly, pH elevation alone is insufficient to account for these effects, as cells exposed to alkaline pH under normocapnic conditions did not accumulate LC3 puncta, and maintaining media pH at 7.4 during 2% CO_2_ exposure still produced robust LC3 puncta formation. Immunoblot analysis and DQ-BSA proteolytic assay further showed that although extracellular pH may modulate the magnitude of the response, low CO_2_ impairs flux independently of pH. While alkaline pH can engage energy-sensing PI3K/AMPK/mTORC1/2 pathways (Kazyken et al., 2023, 2021), our data suggest that the hypocapnia-induced autophagy phenotype reflects additional CO_2_-dependent mechanisms, potentially involving metabolic rewiring, lysosomal dysfunction, or impaired autophagosome–lysosome fusion.

Interestingly, while autophagosome formation is increased, hypocapnia also triggers a robust increase in lysosomal number. RNA-sequencing and functional knockdown studies identified TFE3 as a major mediator of this lysosomal biogenesis response to hypocapnia. Low CO_2_ increased expression of lysosomal and autophagy-related genes, enriched TFE3-associated transcriptional programs, promoted TFE3 dephosphorylation, and drove its nuclear accumulation. Depletion of TFE3 attenuated induction of its target genes such as *Lamp2* and *Neu1*, and reduced the hypocapnia-induced increases in LAMP2 and LC3-II. These data are consistent with previous reports showing that TFE3 and TFEB translocate to the nucleus in response to nutrient and lysosomal stress to expand lysosomal capacity (Napolitano and Ballabio, 2016). However, this compensatory transcriptional response failed to restore degradative function under low CO_2_. Despite enhanced Lysotracker and Lysosensor staining, indicative of lysosomal abundance and acidification, hypocapnic cells exhibited impaired lysosomal proteolytic activity, as evidenced by reduced Cathepsin D maturation and loss of DQ-BSA degradation. Thus, lysosomes formed under hypocapnia appear abundant but functionally compromised, resembling phenotypes observed in lysosomal storage disorders or defects in vesicle fusion and maturation.

A key mechanistic insight from this study is the energetic uncoupling that underlies this flux disruption. Contrary to expectations, hypocapnia did not activate AMPK. Instead, intracellular ATP levels were elevated, phosphorylation of AMPK^T172^ was reduced, and downstream phosphorylation of ACC^S79^ and ULK1^S555^ was suppressed. Since AMPK regulates not only autophagy initiation but also late-stage autophagosome–lysosome fusion via ULK1 and VPS34 complex components (Kim et al., 2011), its suppression may compromise vesicle maturation. Sustained mTORC1 activity, as inferred from persistent pULK1^S757^ and pS6^S240/244^ levels, could further inhibit fusion and lysosome competence. These signaling changes occurred in the absence of exogenous nutrients or growth factors, indicating that CO_2_ tension itself directly modulates metabolic signaling.

Together, these data support a model in which hypocapnia creates a false nutrient sufficiency state, characterized by high ATP, low AMPK, and persistent mTOR activity, that impairs autophagic degradation despite autophagosome induction. Consistent with this model, autophagosome-lysosome fusion was reduced under hypocapnia. Rapamycin restored LC3-lysosome colocalization, implicating mTORC1 activity in the fusion defect; however, rapamycin failed to restore autophagic flux or DQ-BSA degradation. Thus, restoration of spatial proximity or fusion competence alone is insufficient to restore functional autophagy when lysosomal proteolysis remains impaired.

The differential effects of rapamycin, HBSS, and Torin1 further refine this model. Rapamycin selectively inhibited mTORC1 and improved autophagosome-lysosome colocalization but did not rescue terminal degradation. In contrast, both HBSS and Torin1 restored Bafilomycin A1 responsiveness and proteolytic activity under hypocapnia. Since HBSS induces a broad nutrient-stress response that activates AMPK and suppresses mTOR signaling, whereas Torin1 directly inhibits both mTORC1 and mTORC2 (Kim et al., 2011; Thoreen et al., 2009; Liu et al., 2010), these data suggest that complete restoration of autophagic flux requires inhibition of additional signaling nodes beyond mTORC1 alone. The rescue by Torin1 implicates pan-mTOR signaling, including mTORC2. Supporting this possibility, rapamycin increased AKT phosphorylation at S473, consistent with relief of mTORC1-dependent feedback and enhanced mTORC2 signaling. Thus, persistent mTOR pathway activity, particularly involving mTORC2 or mTORC1-mTORC2 crosstalk, may preserve a metabolic state that prevents lysosomal proteolytic recovery under low CO_2_.

These findings have important implications for pathophysiological contexts associated with hypocapnia, including mechanical hyperventilation, panic disorder, ischemic brain injury, and acute lung injury (Laffey and Kavanagh, 2002). In such settings, accumulation of undegraded autophagosomes could exacerbate proteotoxic stress, disrupt organelle quality control, or sensitize cells to secondary insults (Galluzzi et al., 2017). Although this study uses cultured cells and does not directly address *in vivo* consequences, it establishes hypocapnia as a cell-intrinsic stressor capable of altering proteostasis pathways.

Prior studies examining hypercapnia have shown that elevated CO_2_ suppresses autophagy initiation and impairs bacterial clearance in macrophages through Bcl2 and Beclin1-dependent mechanisms (Casalino-Matsuda et al., 2015). In contrast, our findings demonstrate that reduced CO_2_ promotes autophagosome accumulation but impairs autophagic flux. Together, these observations suggest that CO_2_ exerts bidirectional control over autophagy, with hypercapnia inhibiting autophagy initiation and hypocapnia disrupting autophagic completion.

Beyond physiological implications, these observations have important consequences for *in vitro* autophagy studies. Our observations identify CO_2_ tension as an underappreciated yet critical experimental variable. Because autophagy is highly sensitive to metabolic and environmental cues, even modest reductions in CO_2_ can disrupt autophagosome–lysosome fusion and lysosomal degradation, potentially confounding interpretation of autophagy-related assays. Given that CO_2_ levels are often assumed to be stable in cell culture systems yet may fluctuate across incubators or culture conditions, unrecognized variability in CO_2_ tension could introduce artifacts in studies of autophagic flux, lysosomal function, or stress responses.

Several questions remain. First, the molecular mechanism by which low CO_2_ activates TFE3 despite sustained mTORC1 signaling remains unclear and may involves phosphatases, altered lysosomal signaling, or calcium-dependent pathways. Second, the basis for impaired lysosomal proteolysis despite preserved acidification warrants further study and may involve defective hydrolase maturation, altered lysosomal ion homeostasis, impaired trafficking of proteases, changes in lysosomal membrane composition, or altered V-ATPase-associated signaling. Third, the fusion defect merits direct analysis of Rab7, HOPS complex components, SNARE proteins, and lysosomal positioning. Finally, the role of mTORC2 should be examined more directly using genetic or selective pharmacologic approaches. Addressing these questions will clarify how CO_2_ tension interfaces with vesicle trafficking and metabolic control mechanisms.

In conclusion, our study identifies CO_2_ tension as a critical and reversible regulator of autophagic flux, acting through coordinated effects on energy signaling, lysosomal biogenesis, fusion competence, and degradative capacity. These findings expand the physiological relevance of gas signaling and position CO_2_ as a metabolic checkpoint in the control of cellular degradation pathways.

## MATERIALS AND METHODS

### Cell culture and CO_2_ modulation

MEFs cell lines were generated from wild-type embryos as postcoital day-13 and cultured in DMEM supplemented with 10% FBS and 1% penicillin-streptomycin at 37°C. Cells were incubated in standard (10% CO_2_) or hypocapnia (2% CO_2_) conditions using dedicated humidified incubators. CO_2_ levels were confirmed and monitored using a Fyrite CO_2_ gas analyzer. For time-lapse live cell imaging, CO_2_ levels were dynamically adjusted using an on-stage incubation system with real-time gas control. Where indicated, cells were treated with Rapamycin (500 nM, 16 h), Torin 1 (250 nM, 16 h), Bafilomycin A1 (100 nM, 2 h), CCCP (20 μM, 2 h), or HBSS (Hank's Balanced Salt Solution). Media pH was adjusted to 8.0 using 0.33 M NaHCO_3_ and to 7.4 by titration with 1 M HCL prior to addition to cells. pH was continuously monitored and verified using a calibrated pH meter or pH strips before, during, and after each experiment.

### Immunofluorescence and confocal microscopy

For TFE3 localization, cells were plated on glass coverslips. Cells were fixed in 4% paraformaldehyde for 15 min and permeabilized with 0.1% Triton X-100 for 20 min, then incubated with anti-TFE3 (Cell Signaling, 81744S, 1:100) and appropriate secondary antibodies (Thermo Fisher Scientific). Nuclei were counterstained using Fluoromount G™ with DAPI (Invitrogen, 004959-52).

To generate stable cell lines for live-cell imaging, we constructed a puromycin-selectable vector encoding GFP-tagged LC3. Specifically, we used the pMRX-No-HaloTag7-mGFP-LC3-mRFP plasmid (Addgene, #184902) as the backbone and subcloned the IRES-Puro cassette from pMRX-IP-GFP-LC3-RFP-LC3ΔG (Addgene, #84572) into the multiple cloning site to enable antibiotic selection. The resulting construct expresses mGFP-LC3 under a retroviral promoter, followed by an internal ribosome entry site (IRES) driving puromycin resistance. Retrovirus was produced in HEK293T cells using standard packaging vectors, and target cells were transduced and selected with puromycin (2 μg/ml) for 3-5 days to establish stable expression. For all live-cell imaging experiments, only the GFP channel was used to visualize LC3 puncta.

For time lapse live-cell imaging, stably transfected cells were imaged every 5 min on a Stellaris 8 FALCON (Leica) confocal microscope using a 63× oil immersion objective with a stage-top incubation and Okolab heater system. All other live cell images were acquired on Keyence microscope using a 60× oil immersion objective. Lysosomes were labeled using LysoTracker Red DND-99 (Thermo Fisher Scientific, L7528, 75 nM, 30 min). Lysosome numbers were quantified using Keyence analysis software. Colocalization was assessed using Pearson's correlation coefficient in the JACoP plugin in FIJI/ImageJ. Lysosomal proteolytic activity was assessed using DQ-BSA kit (Invitrogen, D12050). Lysosensor Green DND-189 (Thermo Fisher Scientific, L7535, 1 μM, 30 min) was used to visualize lysosomal acidification.

### Immunoblotting

Cells were lysed in CST lysis buffer (20 mM Tris-HCl pH 7.5, 150 mM NaCl, 1 mM EDTA, 1 mM EGTA, 0.1% SDS, 50 mM sodium fluoride, 2.5 mM sodium pyrophosphate, 2 mM beta-glycerophosphate, 1 mM Na3VO4, 10 nM Calyculin A) supplemented with protease inhibitors (Roche, cOmplete™, #11836170001). Protein concentrations were measured using BCA protein assay kit (Pierce), and equal amounts were resolved by SDS-PAGE and transferred to PVDF membranes. Membranes were blocked with 5% milk and probed with primary antibodies against LC3 (Cell Signaling, 12741S), p62/SQSTM1 (Cell Signaling, 23214S), Cathepsin D (Cell Signaling, 69854S), AMPK (Cell Signaling, 2532S), phospho-AMPK^T172^ (Cell Signaling, 2535L), ACC (Cell Signaling, 3662S), phospho-ACC^S79^ (Cell Signaling, 11818S), ULK1 (Cell Signaling, 8054S), phospho-ULK1^S555^ (Cell Signaling, 5869), phospho-ULK1^S757^ (Cell Signaling, 14202S), S6 (Cell Signaling, 2217), phospho-S6^S240/S244^ (Cell Signaling, 2215), S6K (Cell Signaling, 9202), phospho^T389^-S6K (Cell Signaling, 9234), mTOR (Cell Signaling, 2983S), phospho-mTOR^S2448^ (Cell Signaling, 5536L), phospho-AKTs473 (Cell Signaling, 9271), AKT (Cell Signaling, 9272), and VINCULIN (Cell Signaling, 13901S) or ACTIN (Sigma, A5441) as loading controls. HRP-conjugated secondary antibodies (Chemicon) were used and developed with SuperSignal™ West Femto Maximum Sensitivity Substrate (Thermo Fisher Scientific, 34096) and revealed by azure biosystems 300. Densitometry was performed using FIJI/ImageJ and normalized to loading controls.

### ATP quantification

Intracellular ATP levels were measured using the CellTiter-Glo® 3D Cell Viability Assay Kit (Promega, G9683) according to the manufacturer's instructions. Briefly, cells were cultured under indicated CO_2_ conditions and equilibrated to room temperature for 30 min prior to the assay. An equal volume of CellTiter-Glo® 3D reagent was added directly to the culture wells (1:1 v/v ratio), followed by gentle mixing for 5 min on an orbital shaker to ensure complete cell lysis and reagent distribution. The plate was then incubated at room temperature for 25 min to stabilize the luminescent signal. Luminescence was recorded using a SpectraMax iD5 microplate reader. Data were expressed in arbitrary units.

### Gene expression analysis

Total RNA was extracted using RNeasy mini kit (Qiagen, 74106) or Vezol with Ferrobeads Quick Nucleic acid (DNA/RNA) Isolation kit (Ferrotec FB100) and reverse-transcribed using SuperScript III First-Strand synthesis system (Invitrogen, cat. #18080051) or HiScript IV RT SuperMix for qPCR (Vazyme, R423). Quantitative PCR was performed using Taq Pro Universal SYBR Green Master Mix (Vazyme, Q712:03) on the Real-Time PCR System (ViiA7 by lift technologies, AB applied biosystems). Gene expression levels were quantified using 2^dCT method. For each target gene, the CT value was determined and subtracted from the CT value for the housekeeping gene TATA binding protein (*Tbp1*). Prior to primer use, primers were validated using a standard curve generated from a 4-point serial dilution of pooled cDNA to ensure accurate and reproducible quantification across a dynamic range. Amplification efficiency and linearity (R^2^) of each primer pair were verified to fall within acceptable ranges (efficiency between 90-110%, R^2^>0.98). Primer sequences are shown in Table 1.

**Table 1.** qRT-PCR primers

Gene	Forward	Reverse
m*Neu1*	GTAGACACTTTCCGCATCCC	CGATGAAGGCTGTAGAGGAC
m*Tbp1*	CCTTGTACCCTTCACCAATGAC	ACAGCCAAGATTCACGGTAGA
m*Lamp2*	GATGTGCCTCTCTCCGGTTA	ATTGGACTGAACGGCTCCTA

For RNA-sequencing, cells were collected in DNA/RNA Shield (Zymo) and submitted for library preparation and sequencing with Plasmidsaurus. Bioinformatics was performed by Plasmidsaurus according to their published protocol. Samples were processed in two independent experiments; however, batch correction was not able to be performed. Differentially expressed genes, identified from pooling the two batches, were identified as log fold change greater than 2 with an adjusted *P*-value less than 0.05. Pathway and transcription factor analyses were performed using Enrichr analysis for genes significantly upregulated under hypocapnia.

### CRISPR knockdown

Gene knockdown was achieved using CRISPR/Cas9-mediated genome editing. Single guide RNAs (sgRNAs) targeting *Tfe3* gene (mTfe3 gRNA-Fwd CACCGGAGGCGTGAGCGGCGGGAAC, mTfe3 gRNA-Rev AAACGTTCCCGCCGCTCACGCCTCC) were obtained from a previously published study, and cloned into the lentiCRISPR v2, Addgene, #52961), which co-expresses Cas9 and a puromycin resistance cassette. Lentiviral particles were produced by co-transfecting HEK293T cells with the sgRNA-containing lentiCRISPR vector and packaging plasmids (psPAX2 and pMD2.G) using Lipofectamine 3000. Viral supernatants were collected 48 and 72 h post-transfection, filtered through a 0.22 μm filter, and used to transduce MEF cells in the presence of 8 μg/ml polybrene. Following transduction, cells were selected with puromycin (2 μg/ml) for 3 days to enrich for transduced populations. Knockdown efficiency was validated at the protein level by immunoblotting for TFE3 antibody.

### Statistical analysis

All experiments were independently repeated at least three times. Data are presented as mean±s.d. Statistical analyses were performed in GraphPad Prism 9 using unpaired Welch's *t*-test or one-way ANOVA with Welch's *t*-test or two-way ANOVA with Tukey's post hoc test or Fisher's LSD test where applicable. Significance was defined as: *P<0.05, **P<0.01, ***P<0.001, ****P<0.0001, and ns, not significant.

## Supplementary Material



10.1242/biolopen.062414_sup1 Supplementary information
