# Supplementary material for: Autophagic flux blockade under hypocapnia reveals CO2-sensitive regulation of autophagy-lysosome homeostasis
Source: Biol Open. 2026 Jun 30;15(6):bio062414. doi: 10.1242/bio.062414 (PMC13382833; doi:10.1242/bio.062414)
Supplement: Supplementary information [file biolopen-15-062414-s1.pdf]

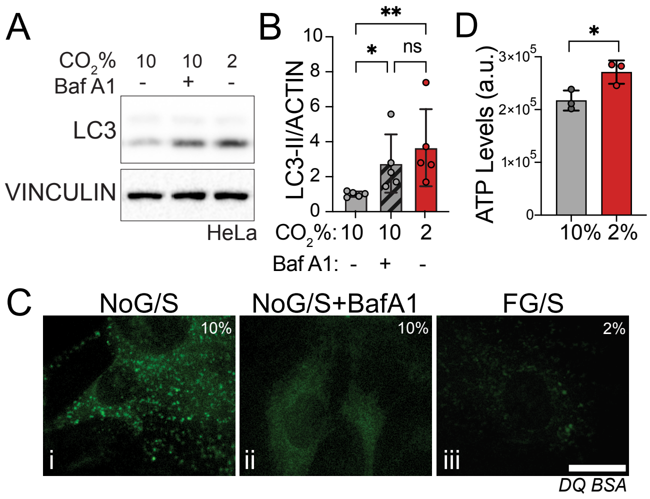

Fig. S1.

(A) Western blot analysis of LC3-II levels in HeLa cells cultured under 2% CO_2 compared to 10% CO_2 in the presence or absence of 100nM BafA1 for 2 hours. **(B)** Quantification of LC3-II levels relative to ACTIN. (n=5; ns = not significant, *p < 0.05, **p < 0.01; One-way ANOVA with Welch's test). **(C)** DQ-BSA (50ug/ml) in cells cultured in glucose- and serum-free media under 10% CO_2 (positive control) (i), in glucose- and serum-free media at 10% CO_2 upon 100 nM Bafilomycin A1 treatment for 2 hours (ii), and in cells cultured in full glucose and serum media at 2% CO_2 (iii). Scale bar: 20 µm. NoG/S: No glucose/serum media, FG/S: Full glucose/serum media. **(D)** Quantification of total ATP levels in cells cultured under 10% or 2% CO_2. **(n=3;** *p < 0.05; Welch's two-tailed t-test).

Biology Open • Supplementary information

Figure 1A

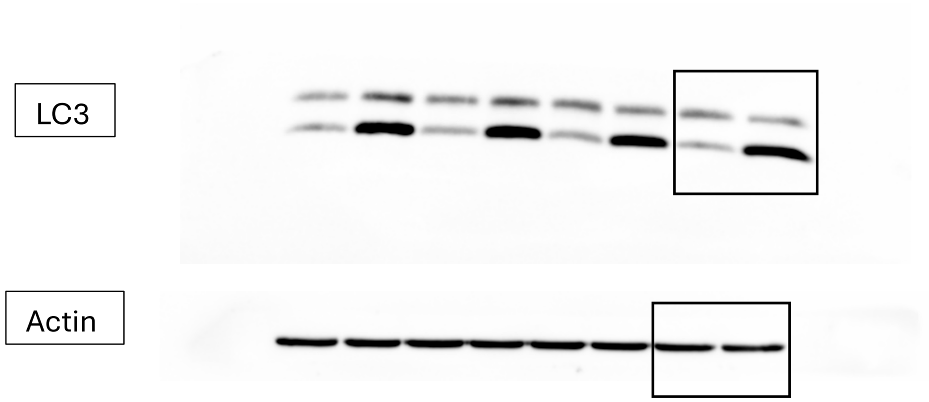

Figure 1B

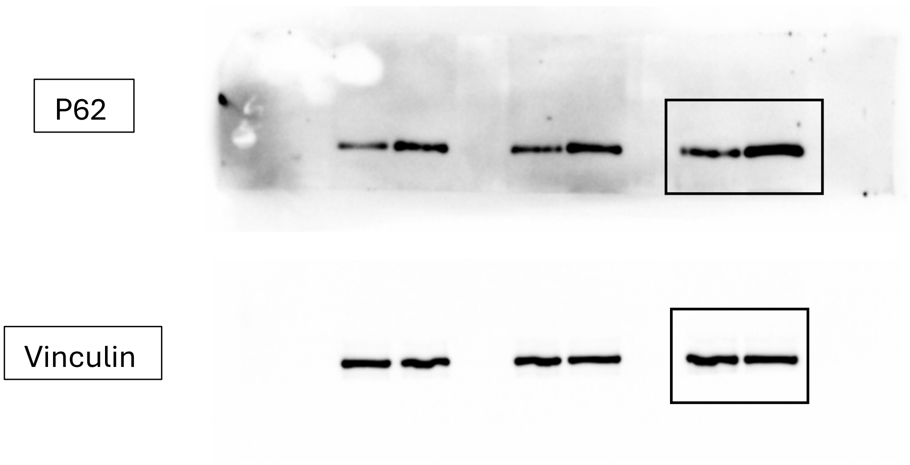

Figure 1E

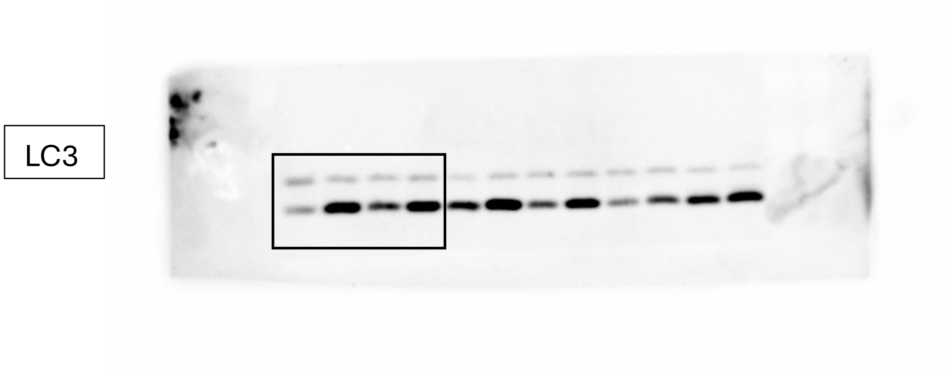

Actin

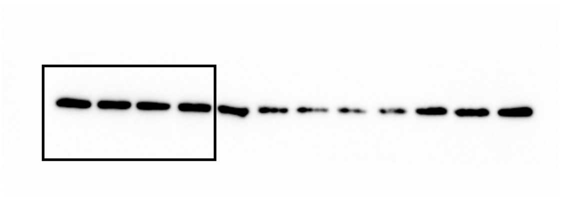

Figure 2B

LC3

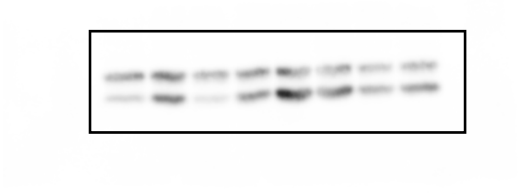

Actin

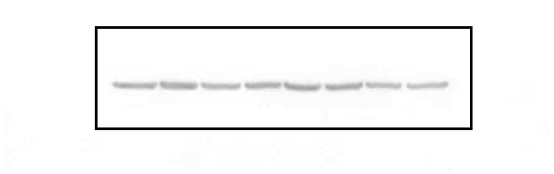

Figure 2E

CTSD

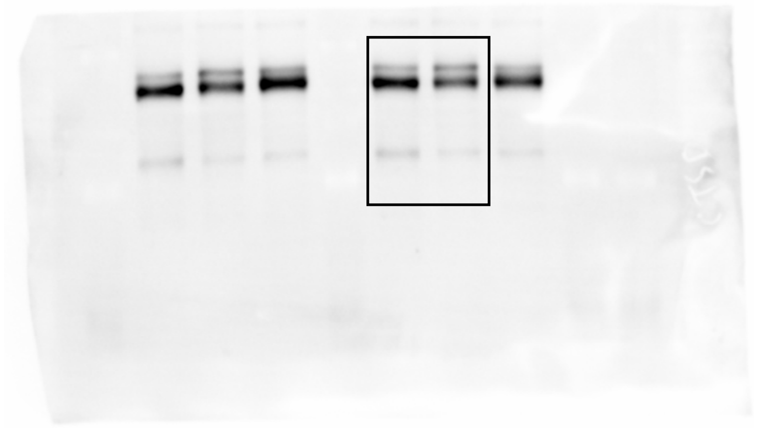

Vinculin

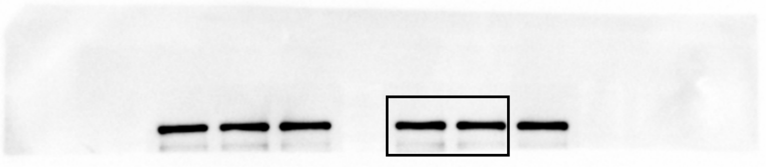

Figure 3A

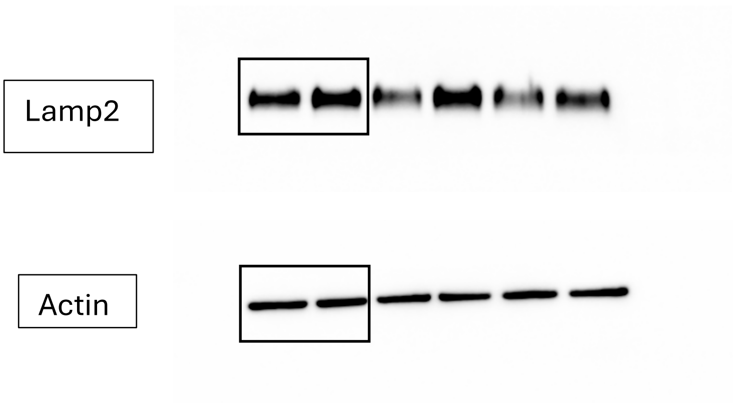

Figure 3E

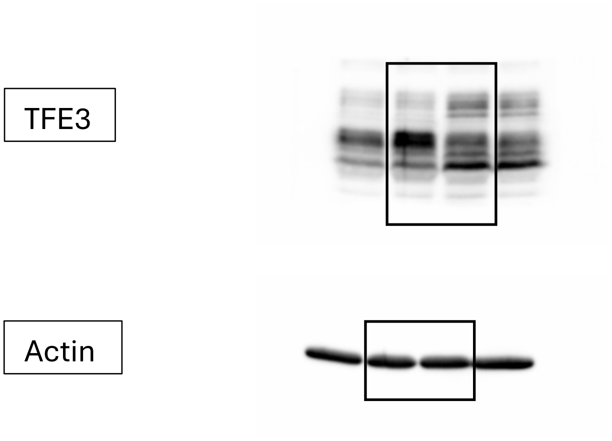

Figure 3G

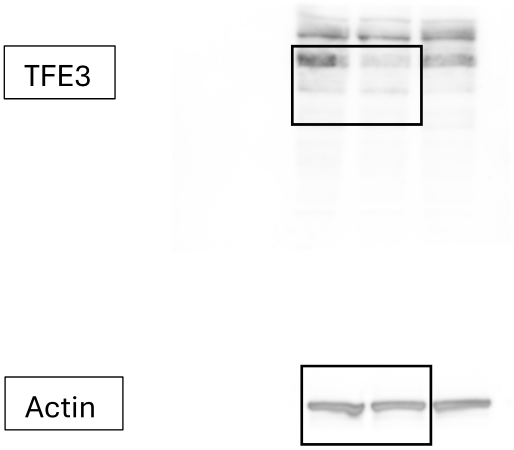

Biology Open • Supplementary information

Figure 3I

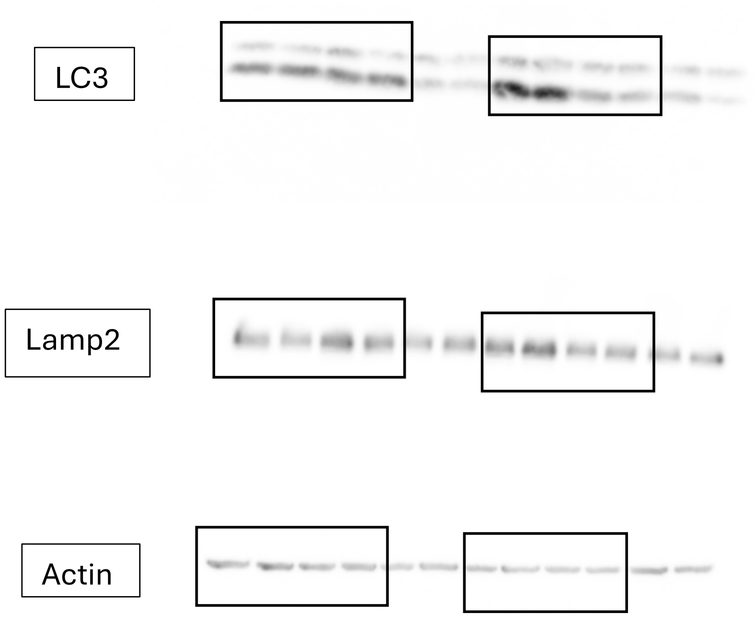

Figure 4C

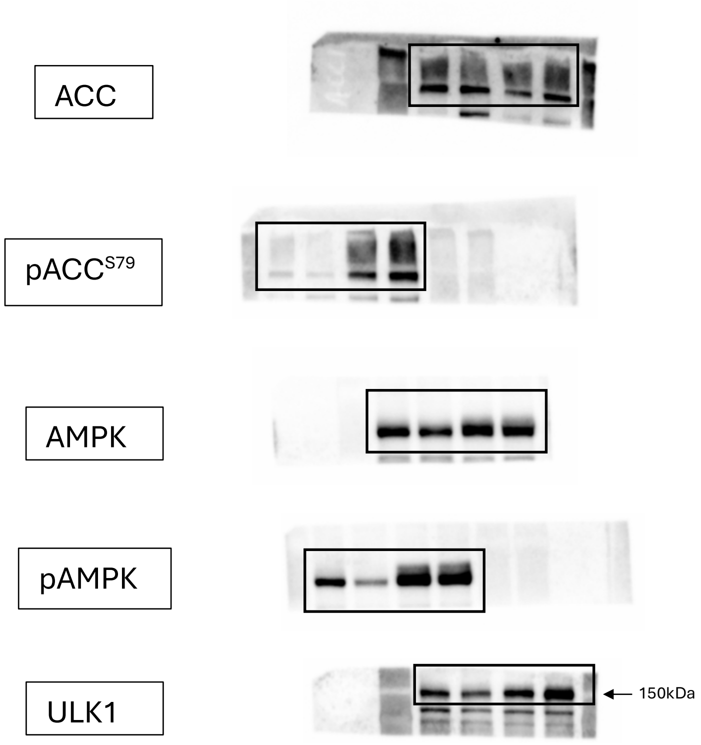

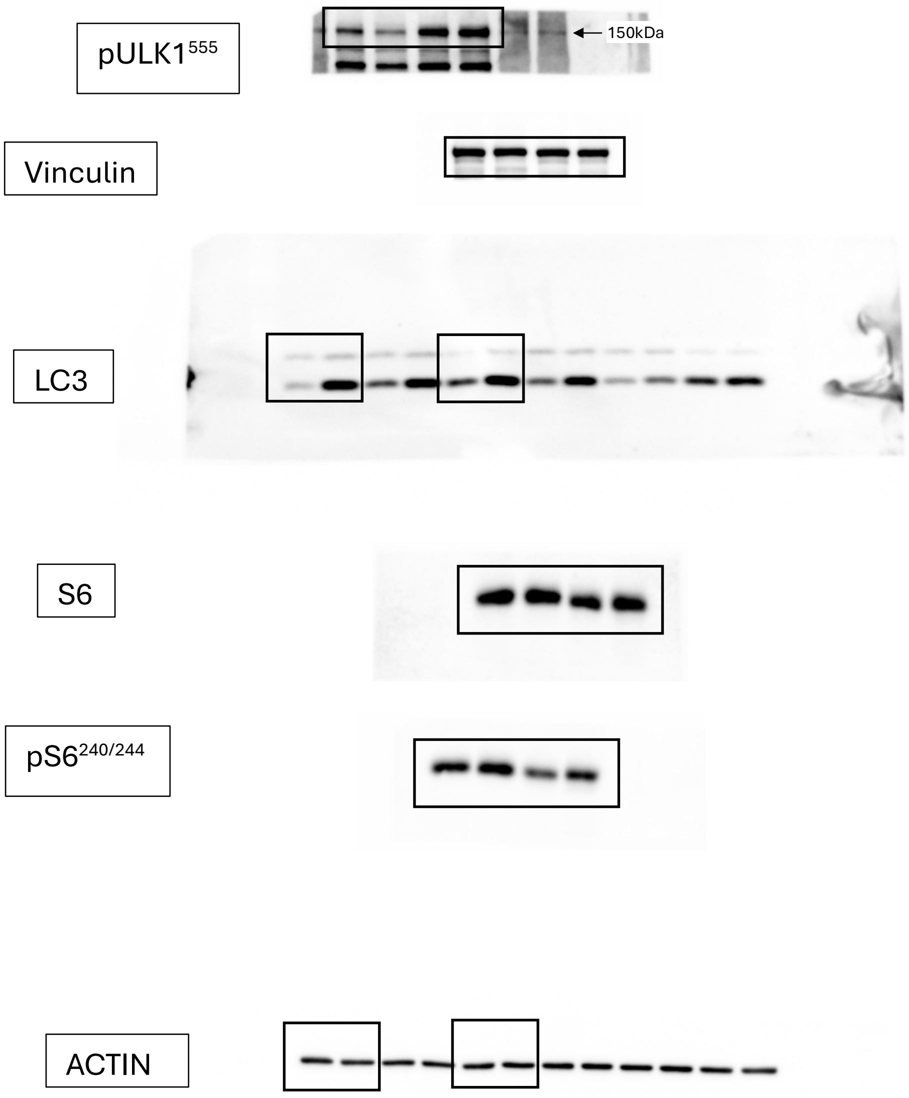

Figure 4D

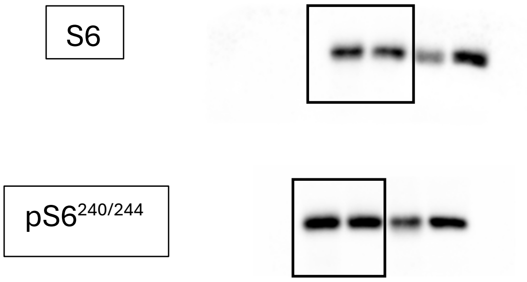

Biology Open • Supplementary information

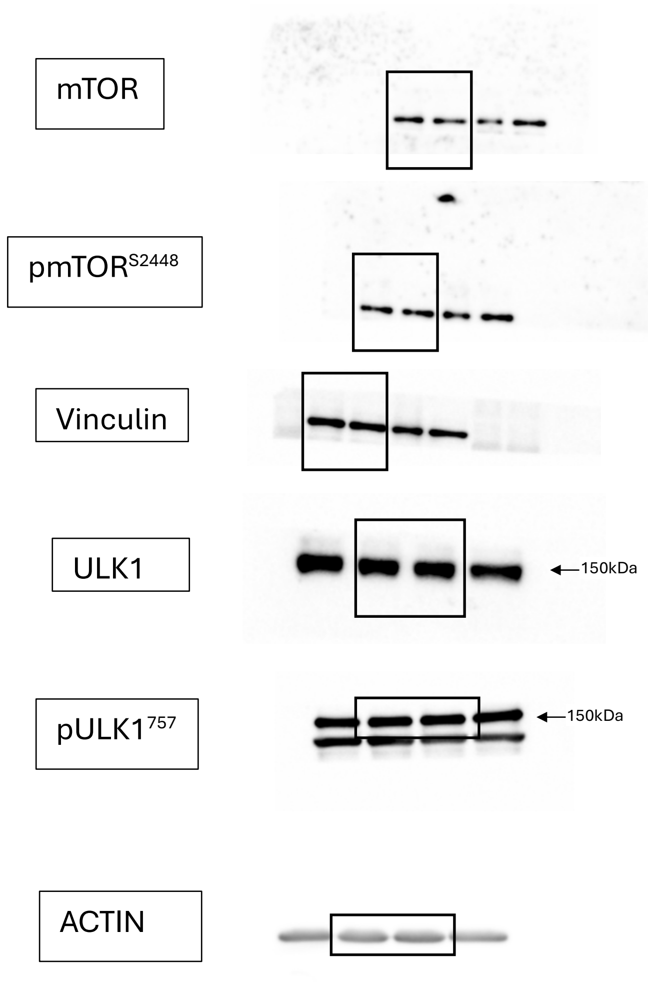

Figure 4E

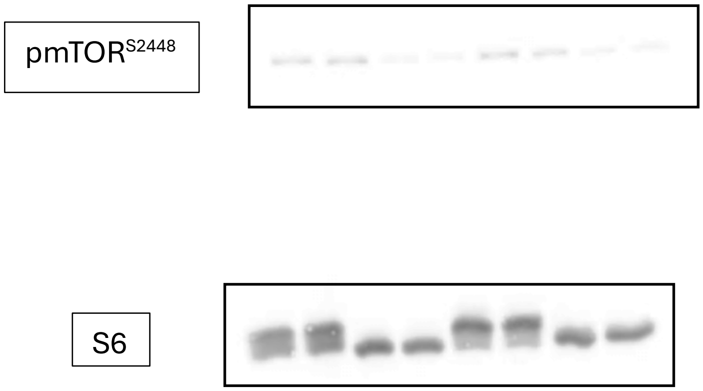

Biology Open • Supplementary information

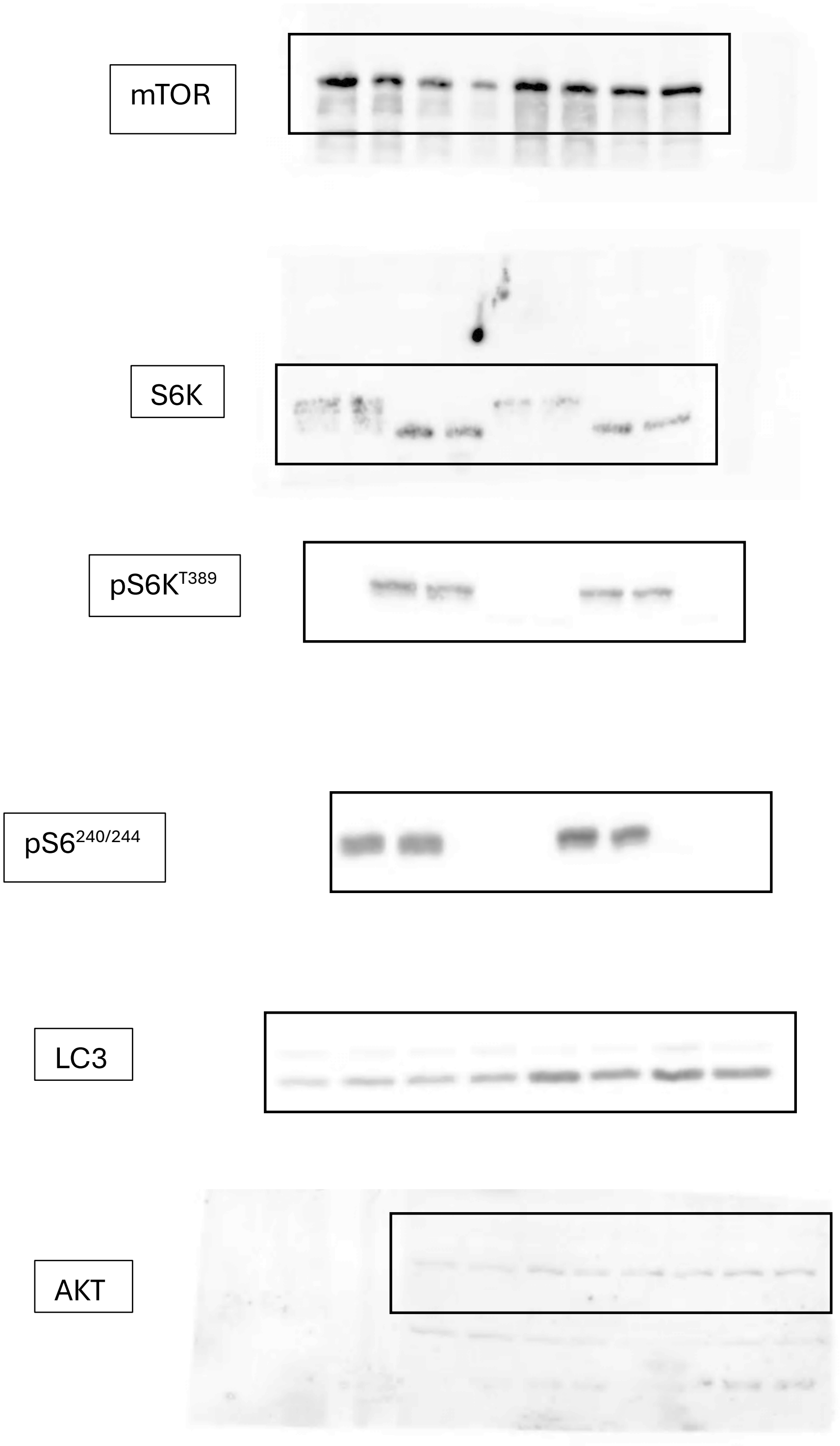
mTOR
S6K
pS6K^{T389}
pS6$^{240/244}$
LC3
AKT

pAKT^{S473}

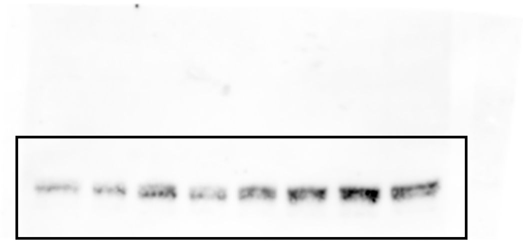

Vinculin

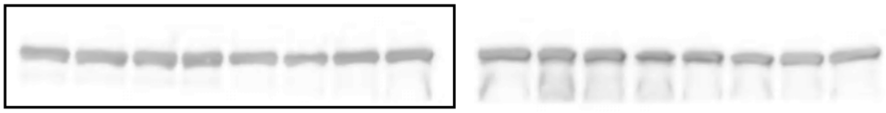

Figure 4F

LC3

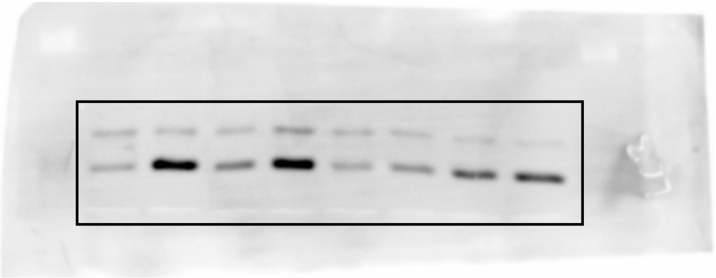

Actin

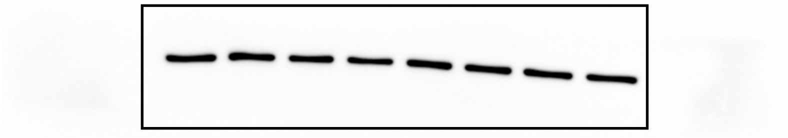

Figure 4H

S6

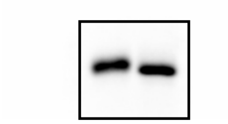

pS6$^{240/244}$

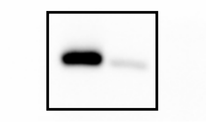

pACC^{S79}

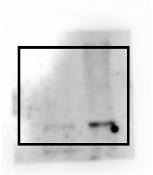

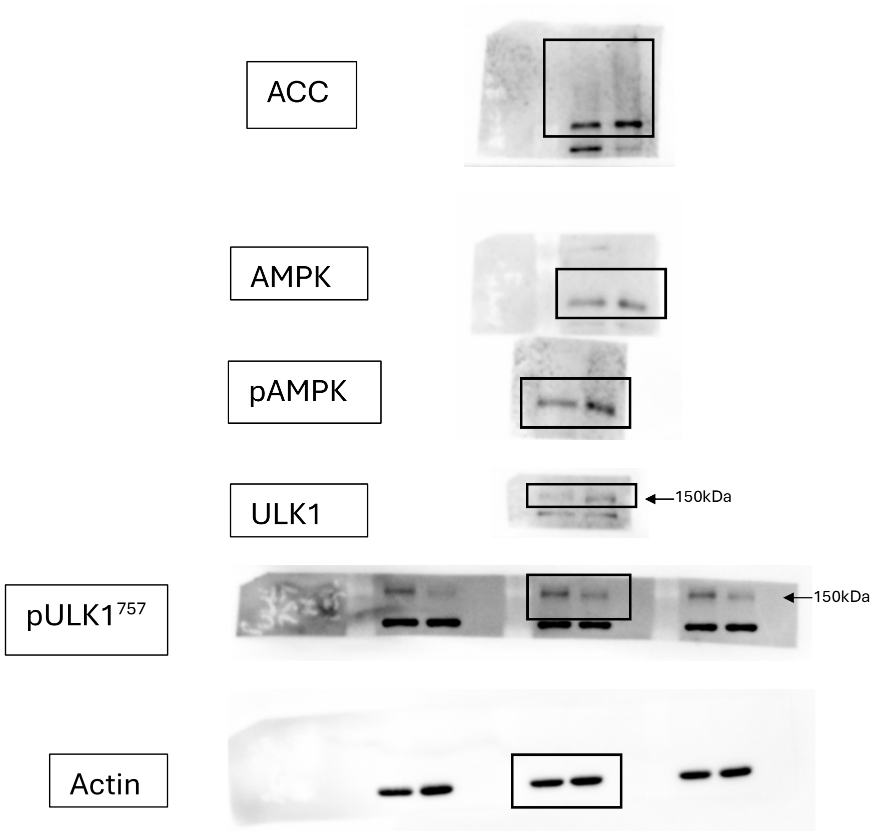

Figure 4I

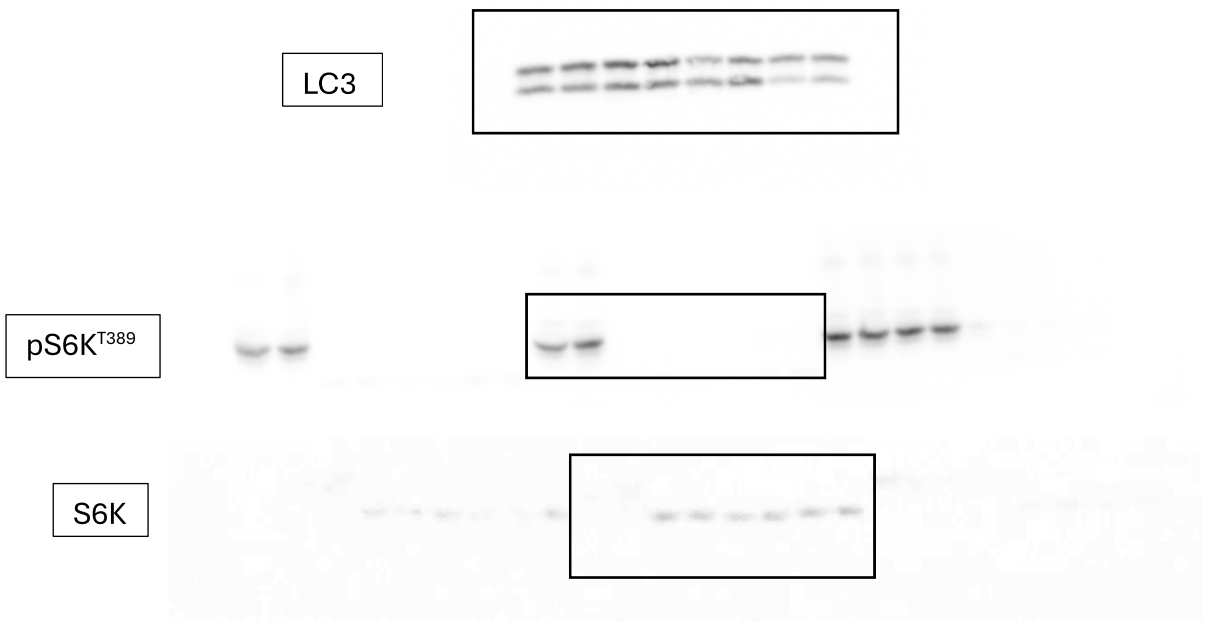

Biology Open • Supplementary information

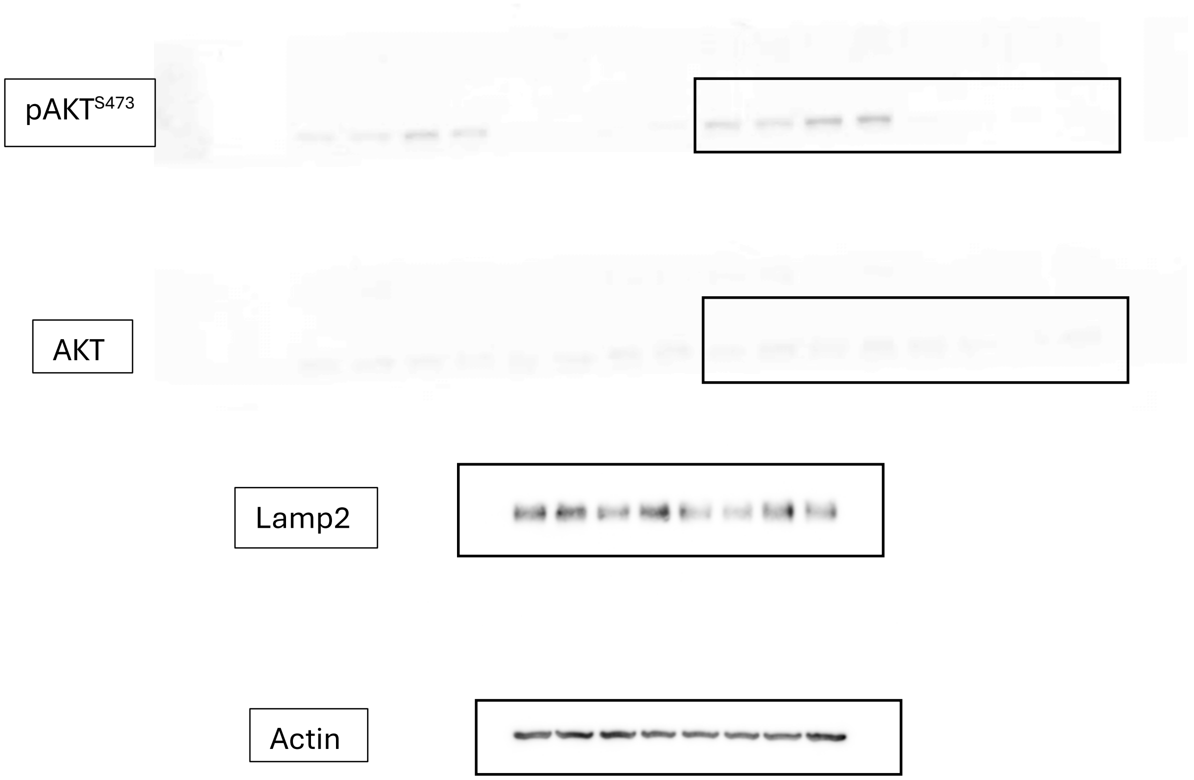

Reviewer Q1

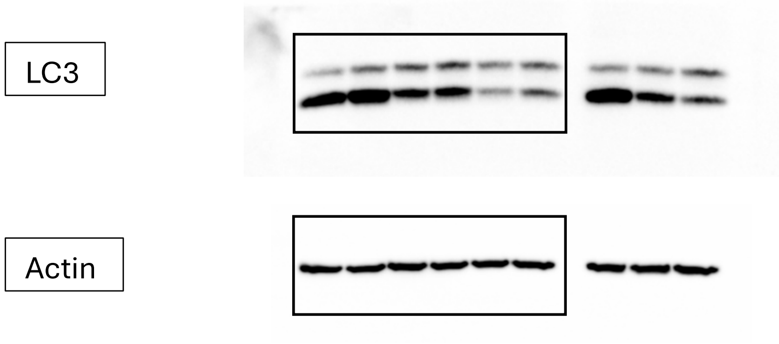

Reviewer Q3

Ponceau

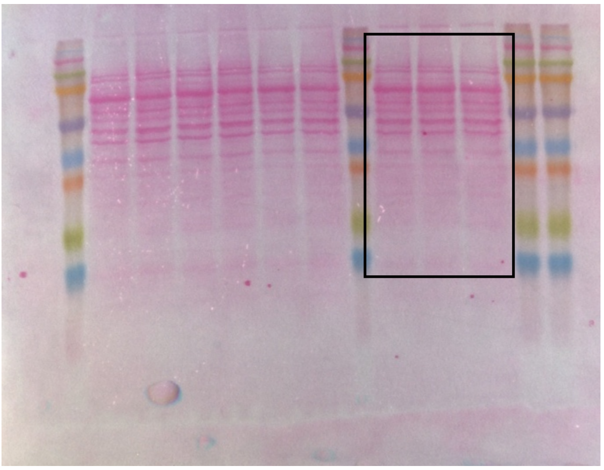

Vinculin

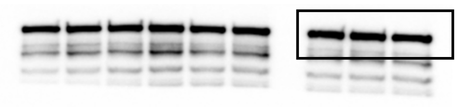

Actin

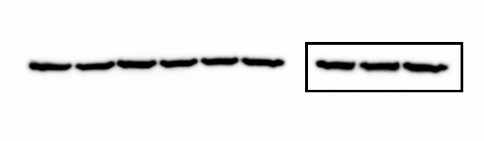

Ponceau

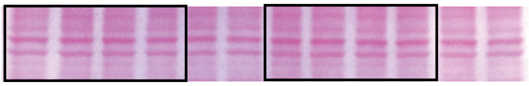

Reviewer2 Q2

LC3

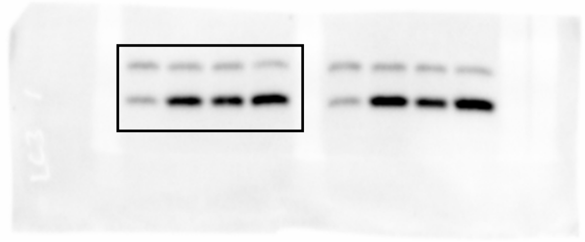

Vinculin

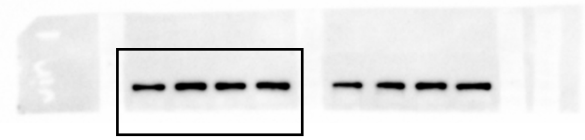

LC3

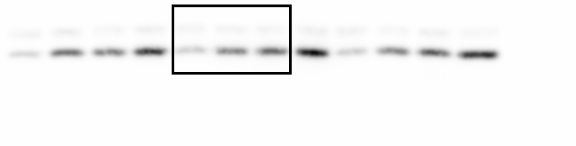

Vinculin

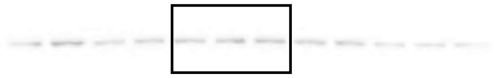

Fig. S2. Blot transparency
