# Supplementary material for: Autophagic flux blockade under hypocapnia reveals CO2-sensitive regulation of autophagy-lysosome homeostasis
Source: Biol Open. 2026 Jun 30;15(6):bio062414. doi: 10.1242/bio.062414 (PMC13382833; doi:10.1242/bio.062414)
Supplement: Supplementary file 1 [file biolopen-15-062414_review_history.pdf]

Autophagic flux blockade under hypocapnia reveals CO2-sensitive regulation of autophagy-lysosome homeostasis

Naghmana Ashraf, Zhen Sun and Jeanine L. Van Nostrand
10.1242/bio.062414

Editor: Luca Scorrano

Review timeline

Original submission:	9 December 2025
Editorial decision:	15 December 2025
First revision received:	30 April 2026
Accepted:	7 May 2026

Original submission

First decision letter

MS ID#: bio.062414

MS Title: Autophagic flux blockade under hypocapnia reveals CO2-sensitive regulation of autophagy-lysosome homeostasis

Authors: Jeanine L. Van Nostrand and Naghmana Ashraf

I have now reached a decision on the above manuscript.

The reviewer reports are shown at the bottom of this email.

As you will see, the reviewers raised a number of substantial criticisms that prevent me from accepting the paper at this stage.

They suggest, however, that a revised version might prove acceptable, if you can address their concerns. If you think that you can deal satisfactorily with the criticisms on revision, I would be pleased to see a revised manuscript. We would then return it to the reviewers.

At this stage, we also ask you to ensure your manuscript complies with our formatting guidelines. Provided you are able to fully address the referees' comments, we are positive about publication of your paper (we accept over 95% of revision submissions) and therefore hope you won't mind any extra work involved in reformatting your manuscript at this point.

Please upload both a 'clean' version of your Word file, along with a highlighted version clearly showing where you have made changes in the revised manuscript. Please avoid using 'Track changes' in Word files as these are lost in PDF conversion.

I should be grateful if you would also provide a point-by-point response detailing how you have dealt with the points raised by the reviewers in the 'Response to Reviewers' box. Please attend to all of the reviewers' comments. If you do not agree with any of their criticisms or suggestions please explain clearly why this is so.

Reviewer 1

Comments for the author

© 2026. Published by The Company of Biologists under the terms of the Creative Commons Attribution License (https://creativecommons.org/licenses/by/4.0/).

Overall comments: In the present manuscript the authors seek to understand the impact of clinically-relevant systemic hypocarbia on autophagy flux. They note that hypocarbia is an often overlooked component of various disease states but that it has been shown to regulate metabolism and cellular pH, suggesting that it may play other roles in regulating cellular function. Overall, the manuscript is very well-written. The introduction is short and to the point and seems to contain sufficient background to set the stage for their study. The methods are clear but may be problematic as the use of immortalized cells cultured in very high CO2 culture media is a questionably choice that muddies their results: physiological CO2 is 4-5% so 10% is too high and 2% is too low…there is no clear control in this study. It would have been preferable to treat primary cells directly with ACSF gassed with different CO2 levels and physiological amounts of bicarb. As designed, the current experiment does not allow the authors to clearly demonstrate that 10% CO2 is "baseline"…this could be the stress condition and going to 2% CO2 could be less stressful than the standard incubation, or it could be stressful in the opposite direction…all of their data could fit with this interpretation and thus their data is equivocal on this point. Otherwise the experimental approach is robust, but the impact of the study is diminished by what is arguably a poor choice of treatment groups. Otherwise, I have some issues with the data collection/interpretation (use of loading controls and presentation of WB data is outdated) and the statistical approach employed, which is not appropriate for multi-level comparisons made in some of the figures.

Major Comments:

1. The authors refer to 10% CO2 as a "physiological level". I do not believe that this is accurate. I believe that DMEM is designed with enough bicarb in it that when cells are incubated in 10% CO2, the resulting shift in pH balance results in an incubation pH that is comfortable for growing cells. By adding less environmental CO2 to the incubator, the cells are being exposed to increasingly basic medium. If the authors are interested in the impact of CO2 as a gassotransmitter then they should treat cells in physiological levels of CO2. I believe systemic blood CO2 in animals is usually in the 4-5% range. So 10% is unphysiologically high and 2% in unphysiologically low. As such, the authors are actually testing the impact of going from super super high CO2 to super low CO2, relative to what cells experience in a body. I encourage the authors to revise their manuscript around this issue because I do not think that their current approach accurately assays physiologically relevant impacts of CO2 in any way. For example, how can they be sure that the "reduced" level of autophagy in 2% CO2 isn't an overshoot from "normal" at 4-5% CO2, and instead there isn't a high level of autophagy in the 10% cells because they are exposed to such unphysiologically high CO2?

2. The authors seem to rule out pH as a cause of the mechanisms they explore, suggesting that their results are largely due to the gassotransmitter role of CO2. But this would have been far clearer if, having established that pH is not changing autophagy, they then balanced their 2% CO2 cells to have the same pH as the 10% CO2 cells. This would have removed this confounding variable and allowed the authors to carefully evaluate the impact of the gassotransmitter function specifically. Having not done this, the results remain muddled. Including experimental replicates of the downstream molecular experiments with pH-treated cells would be important to differentiate between these potential effects. If the authors do not wish to conduct these additional experiments then the text of the manuscript should be carefully revised to recognize the limits of their studies and acknowledge that their conclusions are based on a somewhat large assumption of pH not playing any role in the various downstream pathways they explored.

3. The presentation of the western blot data is not acceptable. The current standard in the field is to provide full, uncut blot images to avoid any concern about manipulation. Loading control scans of Ponceau S or similar should also be supplied for EVERY blot. These can be unwieldy and included in supplementary files. The WB data should also be properly quantified using densitometry and graphed with appropriate statistical treatment. Sample sizes and replicates should be clearly described in the figure legends.

4. Use of loading controls for protein analysis and housekeeping gene for PCR. Do the authors have evidence that their loading gene and protein controls are not changed in different CO2 incubation conditions? Loading controls often change in unexpected ways and their use is becoming outdated. Protein data should be normalized to protein loaded into each well (based on Ponceau scans) and

© 2026. Published by The Company of Biologists under the terms of the Creative Commons Attribution License (https://creativecommons.org/licenses/by/4.0/).

gene analysis should be normalized using the normagene approach. Alternatively, the authors can provide proof the their housekeeping proteins and genes do not change in hypercarbia.

5. Statistical tests are inappropriate in some cases. When comparing between the impact of multiple CO2 levels and drugs, t-tests are not useful. Fig 1F, 2C, and all WB data must be analyzed with a 2-way ANOVA and appropriate post-test.

Reviewer 2

Comments for the author

The manuscript presented by Ashraf and Van Nostrand is reporting one of the adaptive responses engaged by MEF cells to hypocapnia. Specifically, authors showed that in this non-physiological condition, cells exhibit autophagy block, TFE3 nuclear translocation, sustained biogenesis of novel, but barely functional, lysosomes. The overall manuscript is logically organized and it reads well in each part. Moreover, figure panels are well-arranged and the language of the text is polished and accessible for a broad audience, who may not be necessary involved in the field of autophagy. Statistics is rationally used and, for each experiment, sample distribution is showed as dots. The methods are comprehensive, ensuring reliability and rigor in the experimental design. Although the manuscript is of good quality in its current form, there are still areas where the authors' work could be strengthened. Here below, some suggestions and comments.

- The introduction is a bit short and does not include all the necessary literature linking hypocapnia to autophagy as well as to protein-quality control and lysosome metabolic activities.

- It is unclear to this referee if the phenotypes recorded by authors can be generalized to other cell types or are exclusive of the considered cellular models (i.e. MEF cells). Implementing this manuscript by corroborating authors findings in other cell lines would strength the overall message of this manuscript.

- GFP-LC3 puncta in cells exposed 10% CO_2 seem to differ significantly between fig1C and 1Di. Please provide more consistent figures across different experiments.

- The term "lysosomal expansion" is misleading and needs to be replaced by "increased abundance of lysosomes"

- TFE3 protein levels should be flanked alongside test reporting MiTF and TFEB protein abundance and nuclear translocation, in the same experimental conditions.

- Measuring TFEB expression is neither a proxy for TFEB functionality nor sufficient to exclude its participation in lysosomal biogenesis.

- To formally ling TFE3 as a novel sensor guiding lysosomal biogenesis in response to hypocapnia, genetic evidence should be provided. Repeating these simple experiments in MEF TFE3 KO should be prioritized over additional tests.

- Nuclear co-localization of TFE3 signal is only qualitatively reported. Co-localization analysis should be included in the figure to strength authors' data.

- Figure 2E. It is unclear in which form gene expression data are reported. Did authors use relative expression of 2^-ddCt? Data processing and analysis of qPCR info should be clarified and clearly described in M&M.

- Lack of proteolytic activity in lysosomes. The lysosomes generated under hypocapnia conditions appear to lack normal proteolytic activity. This deficit seems to partially arise from the reduced availability of CO_2, which serves as a substrate for the carbonic anhydrase enzyme. When CO_2 levels drop, carbonic anhydrase activity decreases, leading to diminished production of H^+ ions. As a consequence, fewer protons are available for the H^+ pump, thereby limiting the cell's ability to acidify lysosomes and ultimately impairing their proteolytic function. Testing lysosome activity in

© 2026. Published by The Company of Biologists under the terms of the Creative Commons Attribution License (https://creativecommons.org/licenses/by/4.0/).

the presence of CCCP at ph8 should produce the same outcome promoted by hypocapnia. Thus, Lysosome biogenesis seem to be consequence of an inefficient compensative response.

Figure 3A. the labels of the micrographs are not consistent and create confusion. Please replace by providing full details about the experimental conditions tested
HBSS-induced autophagy restoration should be consistent with the experiment presented in figure 3A. Pharmacological block of mTOR activity should mimic the effects of nutrient deprivation. Authors should test whether Rapamycin/Torin1 treatment may be effective in recovering lysosomal impairment. If yes, data presented in figure 3A should be revaluated.

Reviewer's Responses to Questions

Experimental quality
Does each figure have the proper controls?
If 'No', please indicate reasons in Comments for Author box below.
Reviewer #1:
- No

Reviewer #2:
- Yes

Were the data analyzed using appropriate statistical tests?
If 'No', please indicate reasons in Comments for Author box below.
Reviewer #1:
- No

Reviewer #2:
- Yes

Reproducibility
Were experiments performed using adequate number of biological replicates?
If 'No', please indicate reasons in Comments for Author box below.
Reviewer #1:
- Yes

Reviewer #2:
- Yes

Does the methods section provide sufficient detail to permit reproducibility?
If 'No', please indicate reasons in Comments for Author box below.
Reviewer #1:
- Yes

Reviewer #2:
- Yes

Completeness
Are the manuscript's conclusions supported by the data?
If 'No', please indicate reasons in Comments for Author box below.
Reviewer #1:
- No

Reviewer #2:
- Yes

© 2026. Published by The Company of Biologists under the terms of the Creative Commons Attribution License (https://creativecommons.org/licenses/by/4.0/).

Scholarship
Do the authors cite and discuss the merits of data that would argue for and against their conclusion?
If 'No', please indicate reasons in Comments for Author box below.
Reviewer #1:

- Yes

Reviewer #2:

- Yes

Does the manuscript title & abstract accurately reflect the contents of the manuscript, without hyperbole?
If 'No', please indicate reasons in Comments for Author box below.
Reviewer #1:

- No

Reviewer #2:

- Yes

First revision

Author response to reviewers' comments

Reviewer 1:

1. The authors refer to 10% CO2 as a "physiological level". I do not believe that this is accurate. I believe that DMEM is designed with enough bicarb in it that when cells are incubated in 10% CO2, the resulting shift in pH balance results in an incubation pH that is comfortable for growing cells. By adding less environmental CO2 to the incubator, the cells are being exposed to increasingly basic medium. If the authors are interested in the impact of CO2 as a gassotransmitter then they should treat cells in physiological levels of CO2. I believe systemic blood CO2 in animals is usually in the 4-5% range. So, 10% is unphysiologically high and 2% in unphysiologically low. As such, the authors are actually testing the impact of going from super super high CO2 to super low CO2, relative to what cells experience in a body. I encourage the authors to revise their manuscript around this issue because I do not think that their current approach accurately assays physiologically relevant impacts of CO2 in any way. For example, how can they be sure that the "reduced" level of autophagy in 2% CO2 isn't an overshoot from "normal" at 4-5% CO2, and instead there isn't a high level of autophagy in the 10% cells because they are exposed to such unphysiologically high CO2?

We thank the reviewer for raising this important point regarding the physiological relevance of CO_2 levels used in our study. While we agree that arterial CO_2 tension *in vivo* is typically ~5% (~40 mmHg pCO_2), our designation of 10% CO_2 as "physiological" refers specifically to standard *in vitro* tissue culture conditions using DMEM or similar bicarbonate-buffered media, which are optimized for 10% CO_2 to maintain a pH of ~7.4. To clarify, we do not intend to equate 10% CO_2 with *in vivo* systemic CO_2 levels but rather use it as the normocapnic control for cells *in vitro*, where DMEM is formulated with 3.7 g/L sodium bicarbonate, requiring 10% CO_2 for pH balance (**to maintain media pH at ~7.4**). Culturing cells at 2% CO_2 in this medium leads to **media alkalinization**, as also reflected by phenol red.

Furthermore, numerous published studies in the autophagy field have used 10% CO_2 as standard incubation condition for MEFs and other mammalian cells, including: Hung, C. M. *et al.*, Sci Adv 2021, Kazyken, D. *et al., Autophagy 2024, Meley, D. et al.,* **J. Biol. Chem** *2006, Dawar, S. et al., Cell Death Dis 2024, Sakurai, H.T. et al., iScience 2023, Zhang, Y. et al., Cell Rep 2025.* These references illustrate that 10% CO_2 is widely adopted in the field, not as a reflection of *in vivo* gas

© 2026. Published by The Company of Biologists under the terms of the Creative Commons Attribution License (https://creativecommons.org/licenses/by/4.0/).

exchange but to maintain homeostatic pH and nutrient stability in culture. Thus, our experimental design reflects standard autophagy assay conditions, and our comparison of 10% vs. 2% CO_2 is consistent with the existing literature.

Importantly, to address the reviewer's concern that the difference between 10% and 2% CO_2 may not reflect physiological variation, we used intermediate CO_2 conditions (5%) (see blot shown below). These results demonstrate that LC3-II accumulation remains elevated under 5% CO_2 as compared to 10% CO_2, indicating that the observed impairment in autophagy is not merely a rebound from "supra-physiological" 10% CO_2, but a consistent feature of CO_2 reduction below culture-maintained levels.

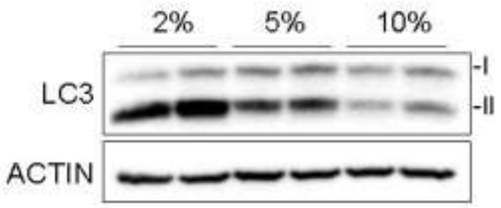

2. The authors seem to rule out pH as a cause of the mechanisms they explore, suggesting that their results are largely due to the gassotransmitter role of CO2. But this would have been far clearer if, having established that pH is not changing autophagy, they then balanced their 2% CO2 cells to have the same pH as the 10% CO2 cells. This would have removed this confounding variable and allowed the authors to carefully evaluate the impact of the gassotransmitter function specifically. Having not done this, the results remain muddled. Including experimental replicates of the downstream molecular experiments with pH-treated cells would be important to differentiate between these potential effects. If the authors do not wish to conduct these additional experiments, then the text of the manuscript should be carefully revised to recognize the limits of their studies and acknowledge that their conclusions are based on a somewhat large assumption of pH not playing any role in the various downstream pathways they explored.

We thank the reviewer for this thoughtful and important point regarding the potential contribution of pH to the phenotypes observed under hypocapnic conditions. We agree that careful separation of CO_2-dependent effects from extracellular pH changes is critical.

To directly address this concern, we performed complementary gain- and loss-of-pH experiments. First, we adjusted the media of cells maintained at 10% CO_2 to pH 8.0, matching the extracellular pH observed at 2% CO_2. Under these conditions, cells failed to form LC3 puncta and did not phenocopy the autophagy-related changes observed under hypocapnia, indicating that extracellular alkalinization alone is insufficient to induce autophagosome accumulation. Conversely, we buffered the media of cells cultured at 2% CO_2 to maintain a neutral pH (7.4). Importantly, stabilizing pH under hypocapnic conditions did not prevent LC3 puncta accumulation or restore sensitivity to Bafilomycin A1, demonstrating that reduced CO_2 levels are sufficient to drive the observed autophagy defects even when extracellular pH is held constant.

In addition, we extended these analyses to downstream lysosomal outcomes. Across varying pH conditions, CO_2 tension was the dominant determinant of lysosomal proteolytic capacity, LC3 accumulation, and refractoriness to Bafilomycin A1, as assessed by Western blotting and DQ-BSA degradation assays. Together, these new data demonstrate that while pH modestly influences the magnitude of LC3 accumulation (as observed in LC3-II levels at low pH at 2% CO2), it is neither sufficient nor required for the autophagic flux blockade induced by hypocapnia.

These findings are now presented in a new Figure 2, and the Results text has been revised to more clearly articulate the scope and limitations of our conclusions. Specifically, we now emphasize that CO_2 regulates autophagy through mechanisms that are largely independent of extracellular pH, while acknowledging that pH may play a modulatory role rather than a primary driving role in this system.

© 2026. Published by The Company of Biologists under the terms of the Creative Commons Attribution License (https://creativecommons.org/licenses/by/4.0/).

3. The presentation of the western blot data is not acceptable. The current standard in the field is to provide full, uncut blot images to avoid any concern about manipulation. Loading control scans of Ponceau S or similar should also be supplied for EVERY blot. These can be unwieldy and included in supplementary files. The WB data should also be properly quantified using densitometry and graphed with appropriate statistical treatment. Sample sizes and replicates should be clearly described in the figure legends.

We appreciate the reviewer's suggestion and agree that transparency and rigor in Western blot presentation are essential. In response, we have now included a supplementary Word file containing **all original, uncut blots** for every figure panel in the manuscript. These are clearly labeled and correspond to each cropped image shown in the main figures.

Regarding loading controls, in our initial experiments, we performed **Ponceau S staining** and compared this with both **ACTIN and VINCULIN** levels across different CO_2 conditions. We observed no significant variability between groups, indicating consistent total protein loading (see blot shown below). Based on these results, and to streamline later experiments, we used **ACTIN or VINCULIN** as the loading controls for subsequent blots. We also ensured that the loading controls used for each blot were run on the same gel and membrane as the target proteins and imaged under identical exposure conditions. These are widely accepted standards and are shown in each relevant panel.

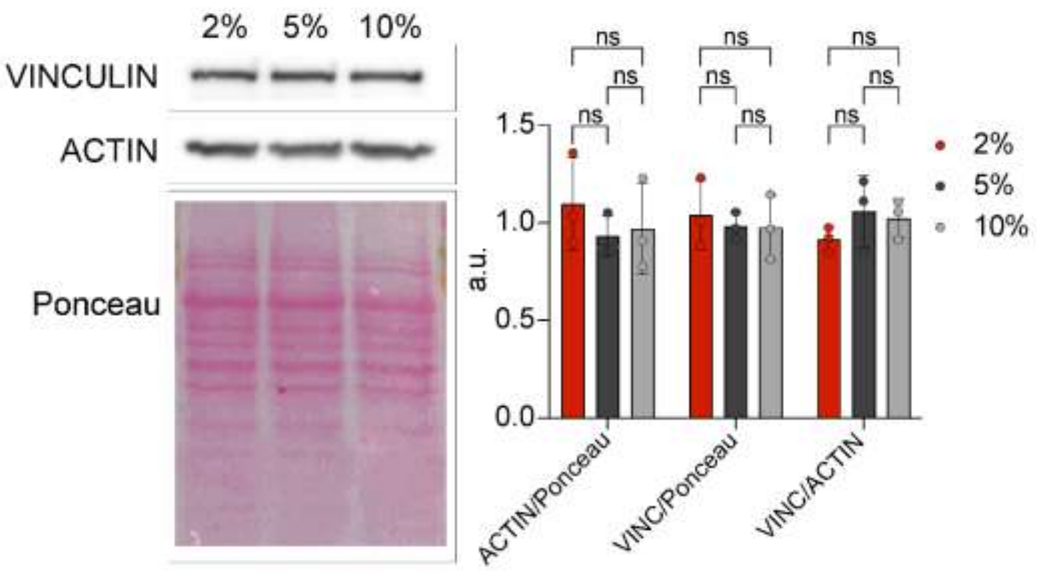

Ponceau S staining in our Tfe3 knockdown cell lines showed comparable total protein levels between 2% and 10% CO_2 conditions, indicating equal loading as shown below.

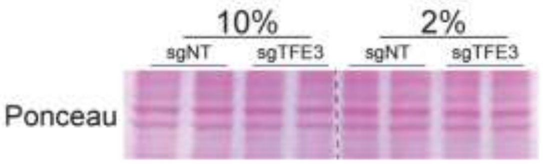

In addition, **immunoblot bands were quantified using densitometry** and we have now included the quantification graphs with appropriate statistical analyses in the figures. Finally, we have updated **figure legends** to clearly describe the **sample size, number of replicates**, and **statistical tests** used. We believe these updates now meet or exceed the current field standards.

4. Use of loading controls for protein analysis and housekeeping gene for PCR. Do the authors have evidence that their loading gene and protein controls are not changed in different CO2 incubation conditions? Loading controls often change in unexpected ways and their use is becoming outdated. Protein data should be normalized to protein loaded into each well (based on Ponceau scans) and

© 2026. Published by The Company of Biologists under the terms of the Creative Commons Attribution License (https://creativecommons.org/licenses/by/4.0/).

gene analysis should be normalized using the normagene approach. Alternatively, the authors can provide proof that their housekeeping proteins and genes do not change in hypercarbia.

We thank the reviewer for raising the point regarding normalization strategies under altered CO_2 conditions. As stated previously, for protein analysis, in our initial experiments we performed Ponceau S staining on membranes prior to immunoblotting and compared total protein loading across CO_2 conditions. These analyses revealed no detectable differences in total protein abundance or transfer efficiency between different CO_2 conditions. In parallel, we assessed commonly used housekeeping proteins (ACTIN and VINCULIN) and observed stable expression across all CO_2 conditions tested, consistent with the Ponceau S results.

For gene expression analysis, we agree that normalization is critical when experimental conditions may broadly affect transcription. Rather than relying on a single arbitrarily chosen housekeeping gene, we validated the stability of our reference gene using RNA-sequencing data generated under the same CO_2 conditions. Specifically, TBP (TATA-binding protein) showed no significant change in expression across 10%, and 2% CO_2 conditions, supporting its suitability as a normalization control in our qPCR analyses. This approach provides an empirical validation of reference gene stability and is conceptually aligned with normagene-based strategies.

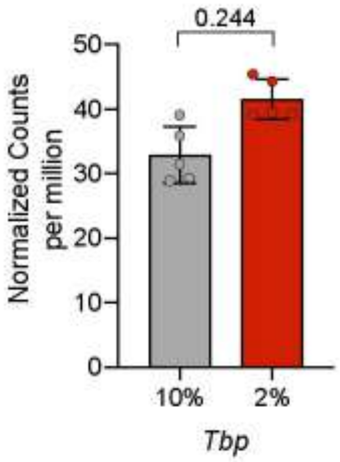

5. Statistical tests are inappropriate in some cases. When comparing between the impact of multiple CO2 levels and drugs, t-tests are not useful. Fig 1F, 2C, and all WB data must be analyzed with a 2-way ANOVA and appropriate post-test.

We have now reevaluated all graphs and performed appropriate statistical tests.

Reviewer 2:

- The introduction is a bit short and does not include all the necessary literature linking hypocapnia to autophagy as well as to protein-quality control and lysosome metabolic activities.

We thank the reviewer for the suggestion to expand the introduction section. We have now incorporated additional background into the link between hypocapnia and protein control and autophagy, which we agree has significantly improved the manuscript.

- It is unclear to this referee if the phenotypes recorded by authors can be generalized to other cell types or are exclusive of the considered cellular models (i.e. MEF cells). Implementing this manuscript by corroborating authors findings in other cell lines would strength the overall message of this manuscript.

We thank the reviewer for raising this important point. To test the generalizability of our findings, we performed LC3 Western blots in additional cell lines, including HeLa cells and lung cancer cell lines, under the same CO_2 conditions used in our primary experiments. Consistent with our results in MEFs, these cell lines also exhibited a significant increase in LC3-II levels under 2% CO_2 (see blot shown below), supporting the conclusion that hypocapnia impairs autophagic flux in a broader

© 2026. Published by The Company of Biologists under the terms of the Creative Commons Attribution License (https://creativecommons.org/licenses/by/4.0/).

cellular context. We have now included the results for HeLa cells in the supplemental figure 1 along with quantification.

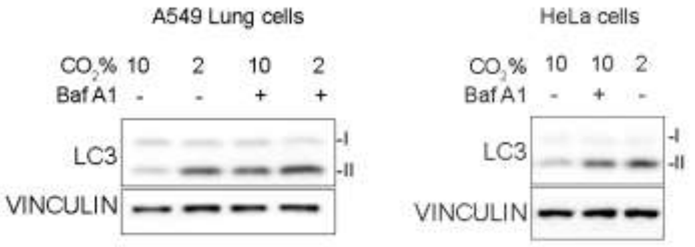

- GFP-LC3 puncta in cells exposed 10% CO_2 seem to differ significantly between fig1C and 1Di. Please provide more consistent figures across different experiments.

We thank the reviewer for pointing out the discrepancy in GFP-LC3 puncta under 10% CO_2 between Fig. 1C and Fig. 1D(i). This variation reflects biological heterogeneity in basal autophagy levels across different cell populations and experimental batches. Even under identical culture conditions (10% CO_2), individual cells can exhibit varying levels of basal autophagosome formation, which has been previously reported in the literature due to factors such as cell cycle stage, metabolic status, and clonal variability.

To address this, we have replaced the representative images in Fig. 1C with new fields that more closely reflect the basal GFP-LC3 puncta levels shown in Fig. 1D(i). This adjustment ensures improved visual consistency across figures without altering the underlying conclusions of the study. The updated images still accurately represent the biological variability and confirm that LC3 puncta increase robustly under 2% CO_2 compared to 10% CO_2.

- The term "lysosomal expansion" is misleading and needs to be replaced by "increased abundance of lysosomes"

We appreciate the reviewer's suggestion and agree that the term "lysosomal expansion" may imply a change in lysosome size rather than number. To avoid ambiguity, we have revised the text throughout the manuscript to use **lysosomal abundance** when referring to the observed increase in lysosomal signal under 2% CO_2 conditions. This change more accurately reflects our data, which are based on lysosomal marker staining and are consistent with an increase in lysosome number rather than volume.

- TFE3 protein levels should be flanked alongside test reporting MiTF and TFEB protein abundance and nuclear translocation, in the same experimental conditions.
- Measuring TFEB expression is neither a proxy for TFEB functionality nor sufficient to exclude its participation in lysosomal biogenesis.

We appreciate the reviewer's suggestion to evaluate MiTF and TFEB protein abundance alongside TFE3 under the same experimental conditions. We attempted to assess MiTF and TFEB protein levels by western blot using multiple validated antibodies from Cell Signaling Technology. However, MiTF protein was undetectable under all conditions tested, and TFEB protein could not be reliably detected except following pharmacologic inhibition of mTORC1, suggesting that basal expression levels of these transcription factors in our model system are very low. Given the inability to detect MiTF and TFEB protein under basal or hypocapnic conditions, we were unable to reliably assess their subcellular localization. In contrast, TFE3 exhibited clear and quantifiable nuclear translocation under low CO_2 conditions.

Consistent with these observations, RNA-seq analysis revealed that *Mitf* and *Tfeb* mRNA levels were 9.3-fold and 17-fold lower, respectively, than *Tfe3*. These marked differences in transcript abundance likely explain our inability to reliably detect MiTF and TFEB proteins by immunoblotting under basal or hypocapnic conditions. These observations indicate that TFE3 is the dominant MiTF/TFE family member expressed in this system, justifying our focus on TFE3 for downstream mechanistic analyses.

© 2026. Published by The Company of Biologists under the terms of the Creative Commons Attribution License (https://creativecommons.org/licenses/by/4.0/).

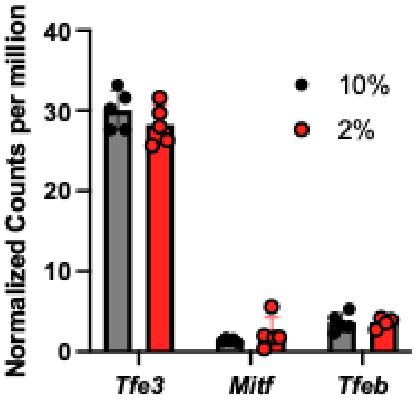

- To formally link TFE3 as a novel sensor guiding lysosomal biogenesis in response to hypocapnia, genetic evidence should be provided. Repeating these simple experiments in MEF TFE3 KO should be prioritized over additional tests.
- Nuclear co-localization of TFE3 signal is only qualitatively reported. Co-localization analysis should be included in the figure to strength authors' data.

We thank the reviewer for these important suggestions. To strengthen the conclusion that TFE3 is a critical mediator of lysosomal response to hypocapnia, we have now incorporated both quantitative imaging and genetic evidence. First, to quantitatively assess TFE3 nuclear localization, we performed **Pearson's correlation coefficient analysis** between TFE3 immunofluorescence and DAPI staining. These data have been added as a new quantitative panel in the corresponding figure with the figure legend updated accordingly (new Figure 3F). This analysis confirms and extends our qualitative observations demonstrating a significant increase in nuclear localization of TFE3 under 2% CO_2 conditions.

Second, to provide genetic evidence for TFE3 function, we generated TFE3-deficient MEFs using CRISPR-mediated targeting of the TFE3 locus. Efficient TFE3 depletion was confirmed by Western blot analysis (new Figure 3G). Using these cells, we assessed transcriptional induction of lysosomal biogenesis genes as well as protein levels of LC3-II and the lysosomal marker LAMP2 (new Figure 3H, 3I). In contrast to control cells, TFE3-deficient cells failed to induce lysosomal gene expression, LC3-II accumulation, or LAMP2 upregulation under hypocapnic conditions. These findings demonstrate that TFE3 is required for the transcriptional activation of lysosomal biogenesis programs and the associated increase in lysosomal abundance in response to low CO_2.

- Figure 2E. It is unclear in which form gene expression data are reported. Did authors use relative expression of 2^-ddCt? Data processing and analysis of qPCR info should be clarified and clearly described in M&M.

We thank the reviewer for raising this important point. The gene expression data in the new Figure 3H were analyzed using the 2^ddCt method using the housekeeping gene TATA-box binding protein (TBP)*Tbp1* as the control. We have updated the Materials and Methods section to clearly describe this quantification approach, including details normalization procedure and data analysis.

- Lack of proteolytic activity in lysosomes. The lysosomes generated under hypocapnia conditions appear to lack normal proteolytic activity. This deficit seems to partially arise from the reduced availability of CO_2, which serves as a substrate for the carbonic anhydrase enzyme. When CO_2 levels drop, carbonic anhydrase activity decreases, leading to diminished production of H^+ ions. As a consequence, fewer protons are available for the H^+ pump, thereby limiting the cell's ability to acidify lysosomes and ultimately impairing their proteolytic function. Testing lysosome activity in the presence of CCCP at ph8 should produce the same outcome promoted by hypocapnia. Thus, Lysosome biogenesis seems to be consequence of an inefficient compensative response.

We thank the reviewer for raising this important mechanistic possibility regarding carbonic anhydrase-dependent lysosomal acidification. We agree that reduced CO_2 availability could, in

© 2026. Published by The Company of Biologists under the terms of the Creative Commons Attribution License (https://creativecommons.org/licenses/by/4.0/).

principle, limit proton generation and impair lysosomal proteolytic activity. However, several lines of evidence from our study argue against impaired lysosomal acidification as the primary cause of the observed loss of proteolytic function under hypocapnic conditions.

First, using LysoSensor-based measurements (new Figure 2C), we consistently observed increased overall lysosomal acidification in cells cultured at 2% CO_2 compared to 10% CO_2. While the number of acidified lysosomes was increased, the fluorescence intensity per lysosome was comparable between conditions and was abolished by Bafilomycin A1, indicating that lysosomes remain acidic and that the V-ATPase remains functional under hypocapnia. These findings are inconsistent with a model in which reduced CO_2 availability limits proton generation and lysosomal acidification.

Second, suppression of lysosomal proteolytic activity under 2% CO_2 was observed despite preserved lysosomal acidity and was not reproduced by manipulation of extracellular pH alone (new Figure 3D). Notably, buffering cells at neutral pH under hypocapnic conditions did not restore DQ-BSA processing, indicating that defective proteolysis cannot be attributed simply to altered proton availability or extracellular alkalinization.

Third, we find that restoring autophagic flux and lysosomal proteolytic function requires conditions that reset cellular metabolic signaling (HBSS or Torin1), rather than conditions that solely modulate pH or acidification (new Figure 4). This suggests that impaired lysosomal function arises downstream of altered nutrient and signaling states rather than from a direct defect in lysosomal acidification machinery.

Finally, while we agree that lysosomal biogenesis under hypocapnia may represent a compensatory response, our data indicate that this response is transcriptionally programmed through TFE3 activation and occurs despite preserved lysosomal acidification (new Figure 3). We therefore conclude that lysosomes generated under low CO_2 are quantitatively increased but functionally compromised due to altered metabolic and signaling context rather than insufficient acidification per se.

We have revised the manuscript to clarify this distinction and to more explicitly state that hypocapnia uncouples lysosomal abundance from degradative function through mechanisms independent of luminal pH.

Figure 3A. the labels of the micrographs are not consistent and create confusion. Please replace by providing full details about the experimental conditions tested

We thank the reviewer for this important suggestion. We have revised the labeling of Figure 3A (new Figure 4A) to ensure consistency and improve interpretability. We have also updated the corresponding figure legends to explicitly define each condition, ensuring that all experimental parameters are clearly stated.

HBSS-induced autophagy restoration should be consistent with the experiment presented in figure 3A. Pharmacological block of mTOR activity should mimic the effects of nutrient deprivation. Authors should test whether Rapamycin/Torin1 treatment may be effective in recovering lysosomal impairment. If yes, data presented in figure 3A should be revaluated.

We thank the reviewer for this important point regarding consistency between HBSS-mediated rescue and pharmacologic inhibition of mTOR signaling. We agree that nutrient deprivation and mTOR inhibition are expected to overlap mechanistically, and we have now clarified this distinction both experimentally and conceptually.

To directly address this concern, we tested the effects of pharmacologic mTOR inhibition on autophagic flux and lysosomal function under hypocapnic conditions. While rapamycin treatment partially restored LC3-lysosome co-localization (new Fig 4A, 4B), it was insufficient to rescue autophagic flux or lysosomal proteolytic activity at 2% CO_2, as evidenced by persistent LC3-II accumulation (new Figure 4E) and lack of DQ-BSA processing (new Figure 4Jii,vi). These data indicate that restoring autophagosome–lysosome fusion alone is not sufficient to overcome the hypocapnia-induced block in lysosomal degradation.

© 2026. Published by The Company of Biologists under the terms of the Creative Commons Attribution License (https://creativecommons.org/licenses/by/4.0/).

In contrast, treatment with Torin1, a pan-mTOR inhibitor targeting both mTORC1 and mTORC2, fully restored autophagic flux (new Figure 4I) and lysosomal proteolytic capacity under hypocapnic conditions (new Figure 4Jiii, vii). Similarly, HBSS treatment, which induces nutrient stress and suppresses both mTOR complexes, rescued autophagic flux and lysosomal function (new Figure 4F, 4G, 4I, 4Jiv,viii). These findings demonstrate that effective restoration of lysosomal degradative activity under low CO_2 requires broader metabolic reprogramming and complete mTOR inhibition, rather than mTORC1 inhibition alone.

We therefore conclude that the results shown in new figure 4A are not inconsistent with the HBSS data but instead reveal a key mechanistic insight: hypocapnia uncouples autophagosome–lysosome fusion from downstream lysosomal degradation, and only conditions that reset global metabolic signaling (HBSS or pan-mTOR inhibition) are sufficient to restore full autophagic flux. We have revised the manuscript to more clearly articulate this distinction.

Second decision letter

MS ID#: bio.062414R1

MS Title: Autophagic flux blockade under hypocapnia reveals CO2-sensitive regulation of autophagy-lysosome homeostasis

Authors: Jeanine L. Van Nostrand, Naghmana Ashraf and Zhen Sun

I am happy to tell you that your manuscript has been accepted for publication in Biology Open, pending our standard publication integrity checks. It was accepted on 7th May 2026.

© 2026. Published by The Company of Biologists under the terms of the Creative Commons Attribution License (https://creativecommons.org/licenses/by/4.0/).
